# Multilocus Analyses Reveal Postglacial Demographic Shrinkage of *Juniperus morrisonicola* (Cupressaceae), a Dominant Alpine Species in Taiwan

**DOI:** 10.1371/journal.pone.0161713

**Published:** 2016-08-25

**Authors:** Chi-Chun Huang, Tsai-Wen Hsu, Hao-Ven Wang, Zin-Huang Liu, Yi-Yen Chen, Chi-Te Chiu, Chao-Li Huang, Kuo-Hsiang Hung, Tzen-Yuh Chiang

**Affiliations:** 1 Kinmen National Park, Kinmen, Taiwan; 2 Endemic Species Research Institute, Nantou, Taiwan; 3 Department of Life Sciences, National Cheng Kung University, Tainan, Taiwan; 4 Institute of Tropical Plant Sciences, National Cheng Kung University, Tainan, Taiwan; 5 Graduate Institute of Bioresources, National Pingtung University of Science and Technology, Pingtung, Taiwan; 6 University Center for Bioscience and Biotechnology, National Cheng Kung University, Tainan, Taiwan; The National Orchid Conservation Center of China; The Orchid Conservation & Research Center of Shenzhen, CHINA

## Abstract

Postglacial climate changes alter geographical distributions and diversity of species. Such ongoing changes often force species to migrate along the latitude/altitude. Altitudinal gradients represent assemblage of environmental, especially climatic, variable factors that influence the plant distributions. Global warming that triggered upward migrations has therefore impacted the alpine plants on an island. In this study, we examined the genetic structure of *Juniperus morrisonicola*, a dominant alpine species in Taiwan, and inferred historical, demographic dynamics based on multilocus analyses. Lower levels of genetic diversity in north indicated that populations at higher latitudes were vulnerable to climate change, possibly related to historical alpine glaciers. Neither organellar DNA nor nuclear genes displayed geographical subdivisions, indicating that populations were likely interconnected before migrating upward to isolated mountain peaks, providing low possibilities of seed/pollen dispersal across mountain ranges. Bayesian skyline plots suggested steady population growth of *J*. *morrisonicola* followed by recent demographic contraction. In contrast, most lower-elevation plants experienced recent demographic expansion as a result of global warming. The endemic alpine conifer may have experienced dramatic climate changes over the alternation of glacial and interglacial periods, as indicated by a trend showing decreasing genetic diversity with the altitudinal gradient, plus a fact of upward migration.

## Introduction

As planet Earth faces rising temperatures linking to escalating anthropogenic greenhouse gas emissions, global changes in its climate are greatly influencing the abundance and distribution of species. In contrast to recent global warming, geological evidence shows that ice ages recurred at regular intervals of 100,000 years followed by a 20,000-year warm period (Milankovitch cycles) during the late Pleistocene [1]. Such climate fluctuations alter the spatial distribution and diversity of species across the world [2]. Further evidence shows latitudinal and altitudinal shifts of species in distribution in response to present-day climate change [3]. In the Northern Hemisphere, during the glacial period, biomes were pushed southward; as the climate warmed, species then expanded northward from the refugia [4]. Apparently, glacial cycles largely shaped the distributional range of the temperate species [2] and determined the spatial apportionment of genetic polymorphisms [5]. Such massive climate changes associated with glaciation often led to large-scale migrations and reductions in population sizes [6].

During the interglacial periods like today, in a subtropical island that were isolated from the adjacent mainland by oceans, with the impacts of increasing temperatures, species previously growing at the mid-elevation habitats were forced to migrate upward and dwell at mountain peaks, resulting in isolated, island-like populations. In contrast, when alpine glaciers grew during glacial periods, previously isolated populations of the alpine species would shift down and connect to each other, forming a meta-population and thus lowering the genetic heterogeneity. Apparently, in contrast to the continental species that experienced north-south migrations over the glacial cycles, the migration of island species, especially those dwelling in alpine regions and hindered by mid-elevation forests, was much more constrained due to oceanic isolation. Accordingly, over glacial-interglacial cycles, the demography of most alpine species on islands periodically fluctuated. Fates of those alpine species were more complicated than expected, given geologically young age of the island with high levels of species diversity/endemism that was originally derived from the adjacent mainland.

According to the geological evidence, the island of Taiwan, part of the island-arc system along the western edge of the Pacific Ocean, first emerged from the water when the Eurasian and Pacific plates collided about 9 million years ago (Ma), but it attained its modern shape only 5–6 Ma [7]. Taiwan’s rugged topography, characterized by hundreds of steep mountains, provides diverse habitats in distinct vegetation zones along the Central Mountain Range [8]. During Pleistocene, a land bridge connected Taiwan with mainland Southeast Asia. Meanwhile, as the global cooling, many plants in the northern continent were forced to migrate into refugia of the southern China and Taiwan [9]. Mainland populations may have become extinct, resulting in the high ratio of endemic relics in the alpine flora of Taiwan. During the late Pleistocene, most alpine peaks in Taiwan are covered with glaciers [10–11], with a range from northern Sheishan, and Nanhutashan, to southern Sanchashan [12], thus severely impacting the distribution of alpine plants. As permanent ice occupies mountain peaks, alpine flora is forced to shift down for survival.

*Juniperus morrisonicola* Hayata (sect. *Sabina*, Cupressaceae), a conifer in the high mountains of Taiwan [13], along the Central Mountain range above 3,000 m in elevation, grows as a dense shrub on mountain peaks and as a tree on the lee slopes. In Taiwan, larger areas of the mountain ranges in the north than in the south foster populations of *J*. *morrisonicola*. For example, the southernmost population comprises fewer than 10 individuals, whereas the populations in northern Taiwan are often large with more than hundreds of thousands individuals. Furthermore, lower annual temperatures in the north would favor this temperate-origin species, partly explaining such a cline along the latitude. In contrast to the species at low elevations, which mostly underwent postglacial demographic expansion [9], *J*. *morrisonicola* is likely to have suffered from the elevating temperatures and shrunk in distribution.

Here we address the following questions by using paternally inherited organellar DNAs [14] and biparentally inherited nuclear DNA (nrDNA) in the conifer of *J*. *morrisonicola*:

1. Did the alternation of glacial/interglacial cycles cause dramatic fluctuations of the effective population size, i.e., recent demographic contraction of *J*. *morrisonicola*?
2. Dwelling at isolated mountain peaks, given hindered gene flow, was the divergence among *J*. *morrisonicola* populations of different mountain ranges enhanced? Did significant genetic differentiation exist among populations of *J*. *morrisonicola* along the Central Mountain range in Taiwan?
3. Shaped by lower levels of impacts of glaciation, did the populations in south maintain more genetic polymorphisms than those in north?

## Materials and Methods

### Populations, sampling and DNA extraction

**Fig 1:**
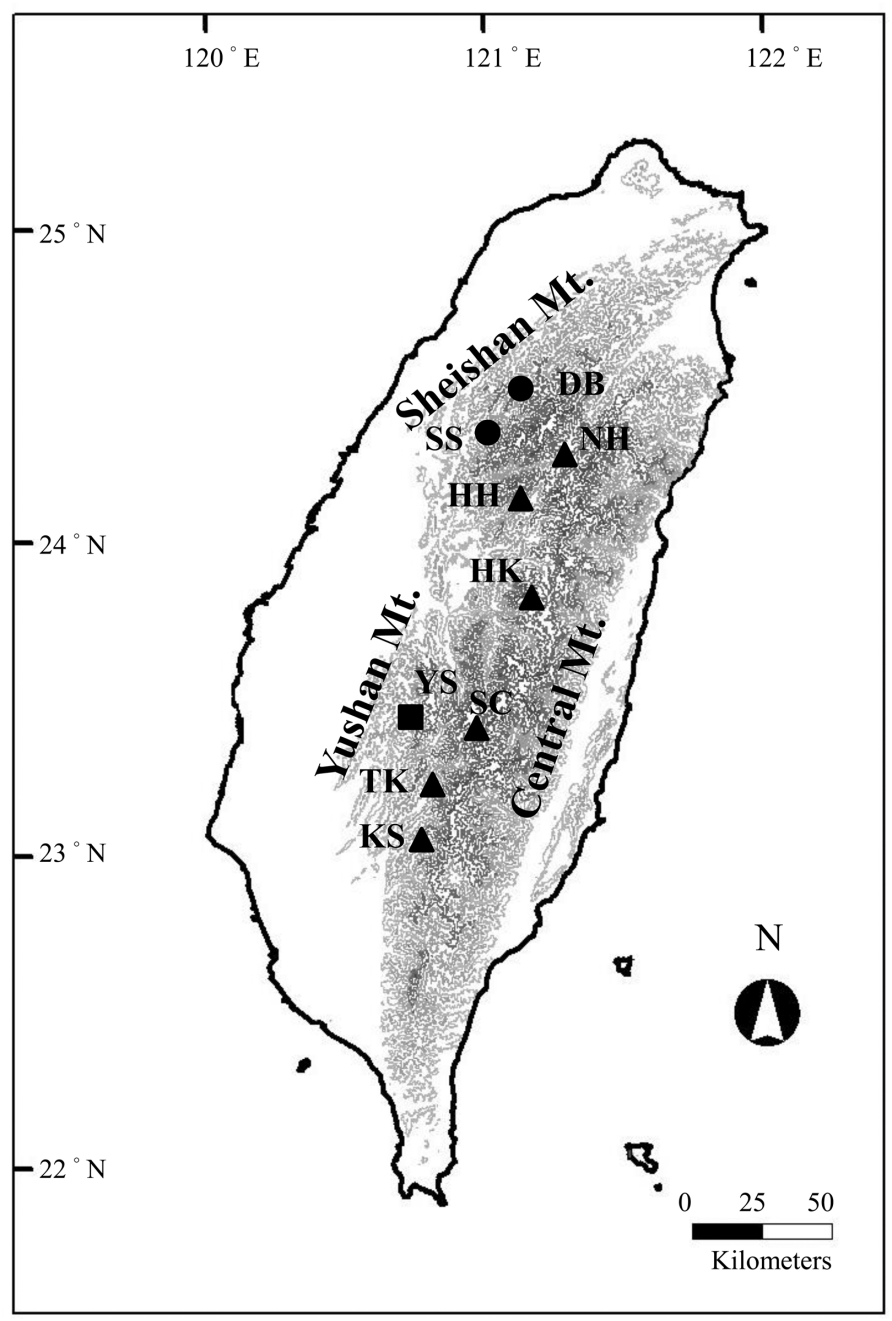
Locations of *Juniperus morrisonicola* populations. Samples were collected from the Sheishan (●), Central (▲)and Yushan (■) Mountain ranges. Abbreviations for populations are given in Table 1.

Nine populations of *J*. *morrisonicola* throughout its entire range of distribution in Taiwan were sampled. In total, 96 individuals were collected in the Sheishan, Central, and Yushan Mountains, which span 3,200 to 3,952 m a.s.l. (Fig 1 and Table 1). No protected or endangered species were involved, so no permit was required to collect tissue in any location sampled in this study. Young, healthy leaves were collected and dried in silica gel for total genomic DNA extraction using the cetyltrimethylammonium bromide procedure [15].

**Table 1:** Locations, codes, and sample sizes (*N*) of sampled populations of *Juniperus morrisonicola* in Taiwan. Sample sizes in parentheses are for the microsatellite analysis.

Species	Location	Coordinate	Altitude (m a.s.l.)	Code	*N*
*Juniperus morrisonicola*					93 (96)
	**Sheishan Mt.**				**18 (19)**
	Dabajashan	24'27"N 121'15"E	3300	DB	11 (11)
	Sheishan	24'23"N 121'14"E	3800	SS	7 (8)
	**Central Mt.**				**66 (62)**
	Hohuanshan	24'08"N 121'16"E	3421	HH	10 (10)
	Hsioukulanshan	23'30"N 121'50"E	3860	HK	9 (7)
	Kuanshan	23'12"N 120'54"E	3600	KS	11 (13)
	Nanhutashan	24'23"N 121'26"E	3700	NH	13 (16)
	Sanchashan	23'18"N 121'01"E	3400	SC	16 (19)
	Takuanshan	23'15"N 120'56"E	3220	TK	7 (7)
	**Yushan Mt.**				**9 (5)**
	Yushan	23'28"N 120'57"E	3952	YS	9 (5)
*Juniperus chinensis* var. *tsukuiensis*	Chingshuishan	24'14"N 121'39"E	2450	JC	1 (0)
*Juniperus formosana*	Sheishan	24'23"N 121'14"E	3800	JF	2 (0)

### Organellar and nuclear DNA analyses

Noncoding spacers *trnS-trnG* and *trnT-trnL* of cpDNA [16] and *coxI* (*Tetraclinis articulata*: EU161447) and *coxIII* (*Juniperus formosana*: EU182939) of mtDNA, and nuclear genes of *Chs*, *Maldehy*, *Myb*, *Needly*, and *Pgi* [17–18] were amplified by the polymerase chain reaction (PCR) using the primers listed in S1 Table. PCR products were electrophoresed on agarose gels, then purified using a gel extraction method. Purified DNA was ligated to a pGEM-T easy vector (Promega), and clones of plasmid DNA were purified using a plasmid mini kit (QIAGEN). Purified plasmid DNA was sequenced on an ABI 3730XL automated sequencer (Applied Biosystem, USA).

### Microsatellite genotyping

Here, 12 variable microsatellites were used for genotyping [19–21]. PCR amplification was carried out in a 50 μL reaction, with forward primers labeled with fluorescent dye. Primer sequences are listed in S2 Table. PCR products were separated with an ABI 3100 automated sequencer (Applied Biosystem, USA), and fragment sizes were assessed using genemapper software version 3.7 (Applied Biosystem, USA).

### Phylogenetic analyses

Nucleotide sequences were aligned with the program Clustal X [22]. Haplotypes of each locus were assigned with the DnaSP v.5.10. Neighbor-joining trees of individual genes were generated using the Kimura 2-parameter model with the program MEGA 5 [23]. Numbers of mutations between DNA sequence haplotypes based on pairwise comparisons were obtained with mega 5 [23], and were used to construct a minimum spanning network with the program minspnet [24].

### Population genetics statistics

Levels of genetic diversity within populations and within species were quantified with measures of nucleotide diversity (*π*) and haplotype diversity (Hd) using DnaSP v5 [25]. Patterns of geographical subdivision and levels of genetic differentiation among mountain systems were estimated hierarchically with DnaSP v5. We also calculated Tajima’s *D* for detecting population growth [26]. A linear regression was also used to investigate the correlation between average genetic diversity across loci and altitude gradients. The significance of the correlation was determined using a regression *F* test in the spss program. The samova program was used to identify groups of geographically adjacent populations of *J*. *morrisonicola* that were maximally differentiated [27]. We performed the analyses based on 100 simulated annealing procedures for *K* values from 2 to 8 and examined maximum indicators of differentiation (*F*_CT_ values).

### Microsatellite fingerprinting analyses

For microsatellites, genetic diversity within populations/mountain systems was assessed by calculating allele number (*A*), observed (*H*_o_) and expected heterozygosity (*H*_e_), and population differentiation (*F*_ST_) with Arlequin v. 3.5 [28]. To detect recent genetic bottlenecks, we conducted a Wilcoxon’s sign rank test on the excess of heterozygosity. Bottleneck tests were performed with the bottleneck 1.2.02 using the infinite alleles model (IAM), stepwise mutation model (SMM), and two-phase model (TPM; 95% SMM and 5% IAM) [29]. We then looked for evidence of genetic structure at a finer resolution by analyzing the data with program structure v. 2.3 [30]. The program structure v. 2.3 uses a Bayesian method to infer the number of clusters (*K*) without using prior information of individual sampling locations. The program was run for *K* = 1 to *K* = 11 clusters with 10 separate runs for each number of clusters to evaluate the consistency of the results. Each run was pursued for 1 million MCMC interactions, with an initial burn-in of 100,000 and an ancestry model that allowed for admixture. The final posterior probability of *K*, Pr (*X*|*K*), was computed using the runs with highest probability for each *K* value [31]. However, as indicated in the structure documentation, Pr (*X*|*K*) usually plateaus or increases slightly after the “right” *K* is reached. Thus, following Evanno et al. [32], we calculated Δ*K*, where the modal value of the distribution is located at the right *K*.

### Demographic fluctuation

For detecting demographic fluctuations, Bayesian skyline plot (BSP) analyses were used to infer historical demographic dynamics of *J*. *morrisonicola* using beast v. 1.7.5 [33]. For BSP analyses, which use standard MCMC sampling procedures calculated from a sample of molecular sequences for estimating a posterior distribution of effective population size without dependence on a pre-specified parametric model of demographic history, multiple loci can be incorporated to estimate the effective population size through time. In this study, we used beast v. 1.7.5 to determine the demographic history that the species experienced in nucleotide sequence loci and microsatellites, respectively. The substitution model for each gene was set as suggested by JMODELTEST v. 2.1.4 [34–35]: GTR for *trnS-trnG*, GTR+I for *Maldehy*, GTR+I+G for *Needly*, HKY for *trnT-trnL*, *coxI*, and *coxIII*, and HKY+I for *Chs*, *Myb*, and *Pgi*. The generation time of *J*. *morrisonicola* was set as 50 years. Linear and stepwise models were explored using a strict clock with substitution rates of 1.94×10^−10^ and 1.12×10^−10^ mutations per site per year for nuclear and organellar loci, respectively [36–37]. For microsatellite loci, a substitution rate of 2×10^−6^ was applied to the genetic analyses. Runs consisted of 50 million generations, with trees sampled every 1000 generations. The BSP was visualized in the program Tracer v1.5 [38], which summarizes the posterior distribution of population size over time. In this study, we also re-analyzed sequences in species of lower elevations or with a wide altitudinal range in Taiwan, including *Castanopsis carlesii* (Hemsl.) Hayata (300–700 m a.s.l.) [39], *Lithocarpus formosana* (Hayata) Hayata (ca. 400 m) and *L*. *dodonaeifolius* (Hayata) Hayata (600–1,200 m) [40], *Cyclobalanopsis glauca* (Thunb.) Oerst. (ca. 500 m) [41], *Cycas taitungensis* C. F. Shen et al. (400–800 m) [42], *Trochodendron aralioides* Siebold & Zucc. (300–2,500 m) [43], *Cunninghamia konishii* Hayata (1,300–2,000 m) [44], *Pinus taiwanensis* Hayata (750–2,500 m) [45], *Michelia formosana* (Kaneh.) Masam. & Suzuki (100–2,200 m) [46], *Machilus kusanoi* Hayata and *M*. *thunbergii* Siebold & Zucc (both ca. 2,300 m) [47], *Euphrasia transmorrisonensis* Hayata (2,200–3,900 m) [48], *Abies kawakamii* (Hayata) Ito (3,000–3,500 m) [49], *Rhododendron pseudochrysanthum* Hayata (3,000–3,900 m) [50] (S3 Table).

**Fig 2:**
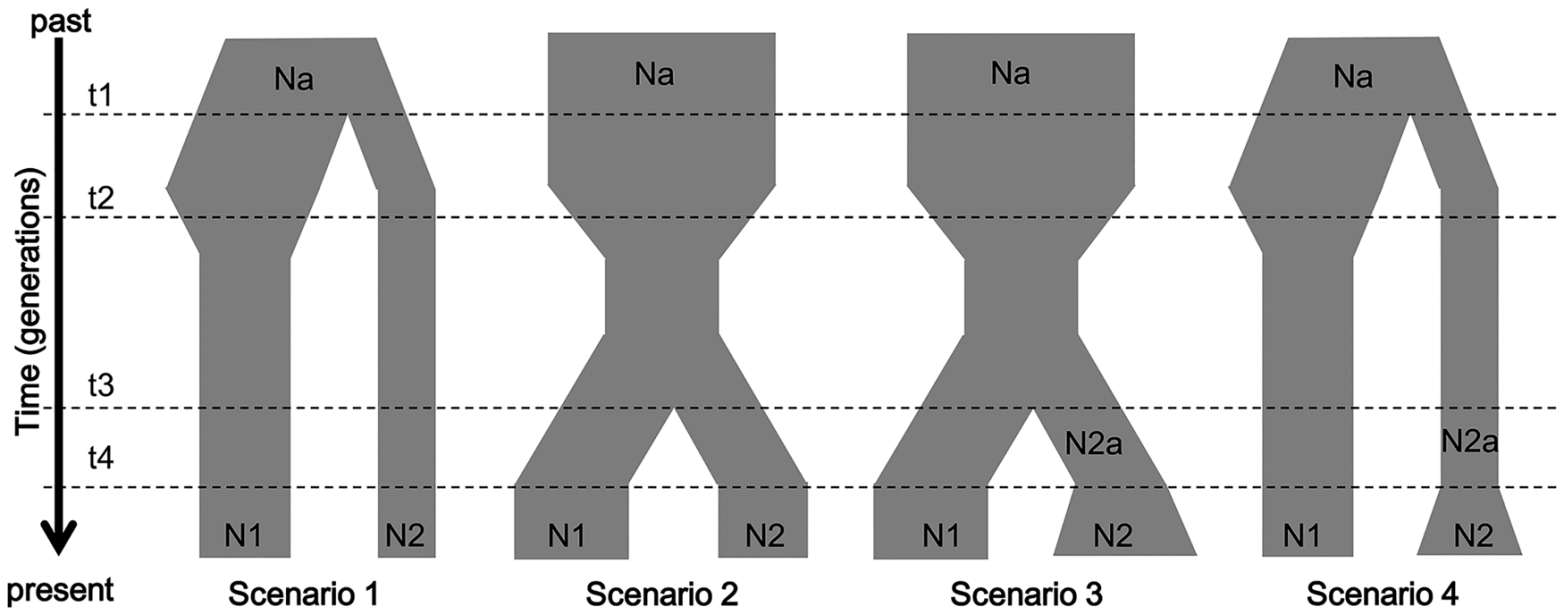
Schematic representation of the four demographic scenarios of the northern and southern populations of *J*. *morrisonicola* in Taiwan. The scenarios were tested by DIYABC analyses. Detailed setting of the demographic parameters was listed in S4 Table. Times and population sizes are not drawn to scale.

To further test the demographic hypotheses suggested by the BSP analyses, an approximate Bayesian computation (ABC) was conducted with the program DIYABC v.2.1.0 [51]. According to the glacial records in Taiwan with the southernmost distribution in Mt. Sanchashan [12], individuals of *J*. *morrisonicola* were thereby divided into the southern group, with populations of TK and KS, both southern from Shanchashan, and the northern group with all other populations. Four hypothetical scenarios based on the timing of population split and the occurrence/absence of recent expansion were tested: (1) the southern group splitting from the northern group prior to the demographic shrinkage of the northern populations, whereas without subsequent expansions (2) the split occurring after the shrinkage without subsequent expansion, (3) the split occurring after the shrinkage, followed by a recent expansion of the southern populations, and (4) the split occurring prior to the shrinkage, followed by a recent expansion of the southern populations (Fig 2). Nucleotide sequences and microsatellite data were analyzed separately. For sequence data, nuclear and organellar loci were assigned to different groups; mutation models were both set as Hasegawa-Kishino-Yano, prior distribution of mutation rate was set as uniform with 1×10^−9^ to 1×10^−6^ per site per generation, and other parameters were set with the default values. For microsatellites, all loci were assigned to a single group with the default values in all settings. The prior distributions for demographic scenarios and the selected summary statistics were detailed in S4 Table. Here, we simulated 1 million and 4.5 million datasets for sequences and microsatellites, respectively. The “pre-evaluate scenario-prior combinations” function in DIYABC was used to confirm if all demographic scenarios generated approximate the observed data. The “compute posterior probability of scenarios” function was used to find the best scenario based on the posterior probability estimated by a logistic regression method.

### Estimation of population demographic parameters

To determine the demographic history that followed the divergence of *J*. *morrisonicola*, we estimated demographic parameters using the program IMa [52]. In this study, the parameters of bidirectional migration rates (*m*_1_ and *m*_2_), and descendent populations (*θ*_1_ and *θ*_2_) were estimated. The HKY and SMM model were applied to sequence data and microsatellites, respectively. The posterior probability densities of these parameters are generated by MCMC simulations, and simulations were run with individual simulations being updated 100,000 times. Within each simulation, we used the procedure to swap among 10 heated chains and observed sufficient swapping rate while the simulation was running. These simulations were carried out using 10 independent runs, with each chain started at a different starting point and initiated with a burn-in period of 100,000 updates. The analyses were considered to have converged upon a stationary distribution if the independent runs generated similar posterior distributions with a minimum ESS of 100. The maximum likelihood estimates for the migration parameters (*m*_12_) were transformed into number of migrants [*M*_12_ = 2*N*_e_ *m*_12_ = *m*_12_(*θ*/2)] was thereby estimated between different mountain ranges.

## Results

### Genetic variations of organellar and nuclear DNA in *Juniperus morrisonicola*

**Fig 3:**
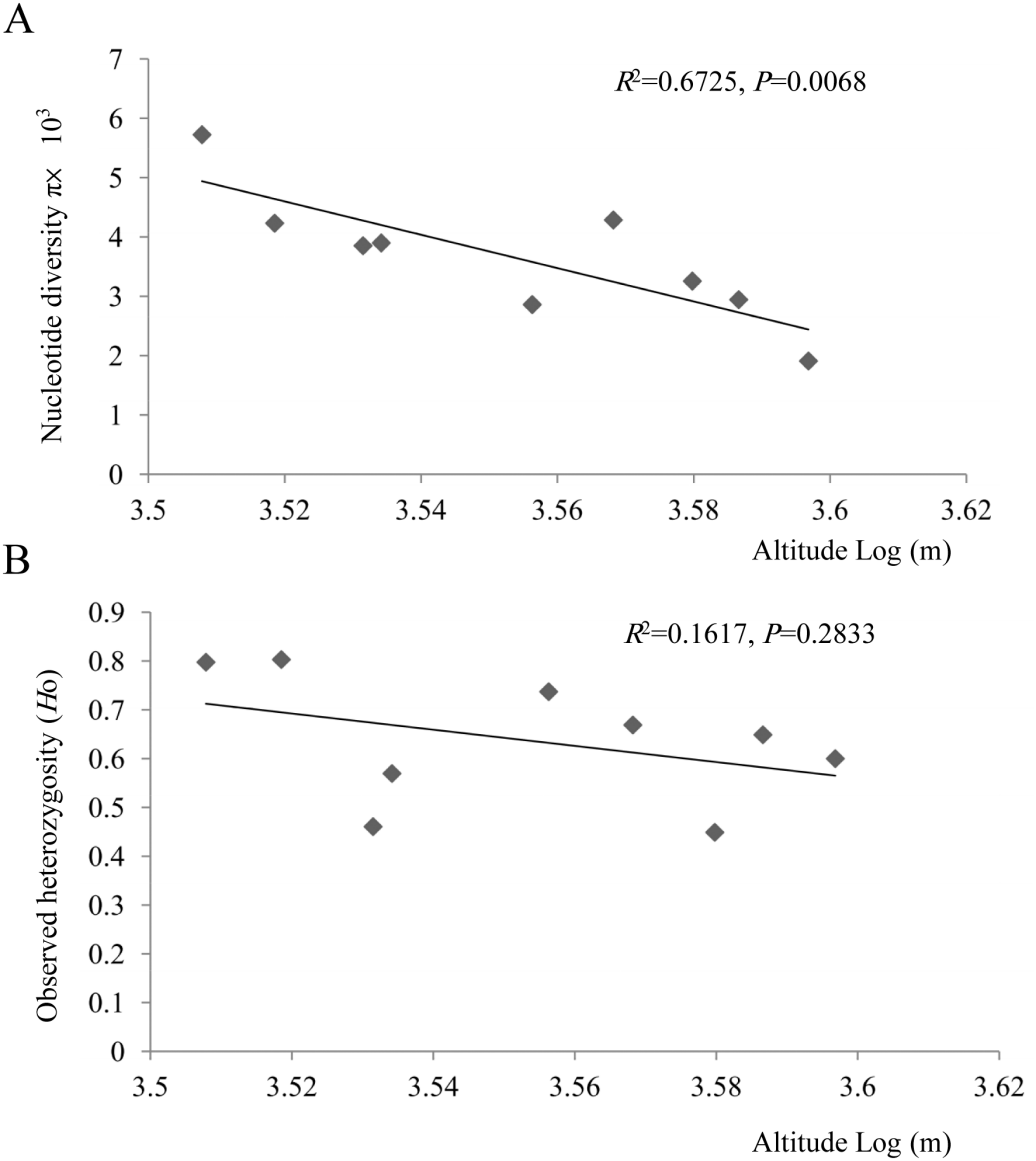
Scatter plot of genetic diversity and logarithmic scales of altitude among populations of *Juniperus morrisonicola*. (A) The scatter plot of nucleotide diversity (*π*) and logarithmic scales of altitude among populations of *Juniperus morrisonicola* based on organellar and nuclear DNAs. (B)The scatter plot of observed heterozygosity (*H*_o_) and logarithmic scales of altitude among populations of *J*. *morrisonicola* based on microsatellites.

In total, 96 individuals were collected from the Sheishan, Central, and Yushan Mountains of Taiwan, which span 3,200 to 3,952 m above the sea level (a.s.l.) (Fig 1 and Table 1). Sequences of 2 chloroplast DNA (cpDNA) spacers, 2 mitochondrial DNA (mtDNA) markers, and 5 nuclear loci were obtained from samples of *J*. *morrisonicola*. The length of the aligned sequences for each locus ranged from 403 to 900 bp (Table 2). Haplotypes with different sequences were deposited in the GenBank database (accession numbers KP727818-KP728023, KT877220-KT877247). In total, 2 and 4 haplotypes for the 2 respective cpDNA markers, 2 haplotypes for the 2 respective mtDNA markers, and 33–51 haplotypes of the nuclear genes were detected in *J*. *morrisonicola*. (S5 Table) For organellar DNA, most populations displayed low haplotype diversities (Hd = 0.000–0.476) except for the KS, SC, TK, and YS populations at the cpDNA. No private haplotypes for *J*. *morrisonicola* were detected at the *trnT*–*trnL* spacer (S5 Table). NH population had the fewest haplotypes, while most populations were fixed at organellar DNA loci. Compared with organellar DNA, nuclear loci had higher levels of nucleotide and haplotype diversities (*π* = 0.00130–0.01283; Hd = 0.427–0.863, Table 2). Nevertheless, the nucleotide diversity of mtDNA was higher for samples from Mt. Sheishan of northern Taiwan than in those from southern Taiwan. Interestingly, altitude (log10 transformed) and average nucleotide diversity of all loci were negatively correlated (*R*^2^ = 0.6725, *P* = 0.0068, Fig 3A). When we considered latitude of populations as a covariate using ANOVA, a significant correlation was also indicated (*R*^2^ = 0.6544, *F* = 5.6805, *P* = 0.0413).

**Table 2:** Summary of nucleotide polymorphisms and neutrality tests. The number of haplotypes is in parentheses. *L*, alignment length; *π*, nucleotide diversity; Hd, haplotype diversity; * *P* < 0.05; ** *P* < 0.01; *** *P* < 0.001.

		cpDNA	mtDNA	nrDNA				
Statistic	Location	*trnS-trnG +trnT-trnL*	*coxI+ coxIII*	*Chs*	*Maldehy*	*Myb*	*Needly*	*Pgi*
***L***		1635	1165	511	532	808	900	403
***Π***								
	All	0.00030	0.00110	0.00130	0.00278	0.00245	0.00706	0.01283
	**Sheishan Mt.**	**0.00000**	**0.00314**	**0.00191**	**0.00251**	**0.00190**	**0.00731**	**0.01264**
	DB	0.00000	0.00203	0.00169	0.00253	0.00185	0.00937	0.01215
	SS	0.00000	0.00494	0.00204	0.00211	0.00088	0.00000	0.01286
	**Central Mt.**	**0.00036**	**0.00064**	**0.00115**	**0.00258**	**0.00247**	**0.00745**	**0.01266**
	HH	0.00024	0.00000	0.00183	0.00223	0.00249	0.00781	0.01269
	HK	0.00015	0.00019	0.00138	0.00236	0.00227	0.00215	0.01210
	KS	0.00036	0.00000	0.00132	0.00167	0.00234	0.00113	0.01320
	NH	0.00000	0.00000	0.00080	0.00241	0.00168	0.01169	0.01342
	SC	0.00041	0.00121	0.00106	0.00341	0.00304	0.00741	0.01034
	TK	0.00076	0.00295	0.00038	0.00176	0.00133	0.01050	0.01333
	**Yushan Mt.**	**0.00040**	**0.00000**	**0.00099**	**0.00428**	**0.00276**	**0.00162**	**0.00133**
	YS	0.00040	0.00000	0.00099	0.00428	0.00276	0.00162	0.00133
**Hd**								
	All	0.427(5)	0.124(4)	0.427(33)	0.764(40)	0.829(51)	0.863(44)	0.856(39)
	**Sheishan Mt.**	**0.000**	**0.307**	**0.602**	**0.778**	**0.769**	**0.807**	**0.781**
	DB	0.000 (1)	0.182(2)	0.593 (8)	0.727 (9)	0.758(10)	0.871 (12)	0.775 (9)
	SS	0.000 (1)	0.476(2)	0.610 (6)	0.738 (5)	0.267 (4)	0.702 (4)	0.805 (7)
	**Central Mt.**	**0.495**	**0.089**	**0.386**	**0.746**	**0.816**	**0.865**	**0.868**
	HH	0.356(2)	0.000 (1)	0.414 (6)	0.745 (9)	0.855 (11)	0.899 (14)	0.809 (7)
	HK	0.222(2)	0.222(2)	0.590 (6)	0.712 (8)	0.795 (8)	0.806 (8)	0.815 (9)
	KS	0.545(2)	0.000 (1)	0.383 (7)	0.460 (5)	0.831 (10)	0.498 (5)	0.801 (11)
	NH	0.000 (1)	0.000 (1)	0.286 (6)	0.756 (7)	0.698 (7)	0.880 (14)	0.856 (13)
	SC	0.575(3)	0.118(2)	0.406 (8)	0.824 (15)	0.846 (17)	0.925 (16)	0.879 (15)
	TK	0.714(4)	0.286(2)	0.186 (3)	0.600 (6)	0.895 (8)	0.833 (6)	0.895 (9)
	**Yushan Mt.**	**0.565**	**0.000**	**0.339**	**0.778**	**0.786**	**0.615**	**0.860**
	YS	0.565(3)	0.000 (1)	0.339 (5)	0.778 (7)	0.786 (6)	0.615 (8)	0.860 (9)
**Tajima's *D***								
	All	-0.83892	-1.35853	-2.416*	-2.039*	-2.144*	-1.342	0.397
	**Sheishan Mt.**	-	-0.13722	-1.96130*	-1.28267	-0.60960	-0.87370	1.12583
	DB	-	-2.04601**	-1.462	-0.945	-0.716	-0.482	2.100*
	SS	-	0.90750	-1.204	0.031	-1.019	-	0.885
	**Central Mt.**	-0.77068	-2.08252*	-2.46756***	-2.04396*	-2.18370**	-1.14763	0.97643
	HH	0.01499	-	-2.15*	-1.482	-0.887	-1.149	1.493
	HK	-1.08823	-1.08823	-1.302	-1.847*	-0.682	0.145	1.170
	KS	1.44272	-	-2.268*	0.456	-0.722	0.797	0.907
	NH	-	-	-1.689	0.860	-0.506	1.269	1.386
	SC	0.09457	-2.26021**	-1.937*	-1.917*	-1.788	-1.321	0.485
	TK	-0.30187	-1.63349*	-1.514	-0.990	1.051	0.745	1.051
	**Yushan Mt.**							
	YS	-0.58325	-	-1.381	-0.663	-0.458	1.014	1.010

### Microsatellite diversity within populations

Estimates of genetic diversity in 12 microsatellite loci varied across populations of *J*. *morrisonicola* (Table 3). The average number of alleles across populations ranged from 5.167 to 10.417. NH population in the Central Mountain range had the most alleles per locus. *H*_o_ and *H*_e_ varied with a range of 0.44891–0.80303 and 0.78804–0.87641, respectively (Table 3). DB population had the highest genetic diversity, whereas SS population had the lowest level of genetic diversity. Significant departures from Hardy–Weinberg equilibrium were detected, indicating a lack of heterozygosity in all populations, except for DB and TK populations (Table 3). As with the sequence data, a negative correlation between elevation and *H*_o_ was also found in the microsatellites (*R*^2^ = 0.1617, *P* = 0.2833, Fig 3B). Moreover, the program bottleneck was used to detect recent genetic bottlenecks. For the SMM, the Wilcoxon’s test detected a recent bottleneck footprint in DB, KS, TK, and YS populations. Under the TPM, the Wilcoxon’s test revealed recent demographic bottlenecks with significant *P* values in all populations, except for SS and HK (Table 3), whereas the IAM showed recent demographic bottlenecks in all populations.

**Table 3:** Comparison of genetic diversity and Wilcoxon test statistics at 12 microsatellite loci in 9 populations of *Juniperus morrisonicola* in Taiwan.

					Fisher’s exact test	*P* values of Wilcoxon test		
Population	*A*	*H*_o_	*H*_e_	*F*_IS_	*P* values	IAM	TPM	SMM
**Sheishan Mt.**	10.250±2.261	0.65659±0.25396	0.86894±0.05023	0.22872	0.00000	**0.00012**	**0.00305**	0.33862
DB	8.917±2.021	0.80303±0.27364	0.87534±0.07416	0.03697	0.30791	**0.00012**	**0.00037**	**0.03857**
SS	5.583±1.443	0.44891±0.35994	0.78804±0.09797	0.50296	0.00000	**0.00403**	0.08813	0.21191
**Central Mt.**	16.500±5.179	0.62702±0.23089	0.89508±0.03563	0.30200	0.00000	**0.00012**	**0.00061**	0.63330
HH	7.750±1.545	0.56948±0.24145	0.87274±0.03949	0.44797	0.00000	**0.00122**	**0.02612**	0.06470
HK	6.917±1.832	0.64881±0.28968	0.85233±0.10077	0.29577	0.00097	**0.01050**	0.25977	0.44922
KS	9.000±1.954	0.73718±0.31709	0.87641±0.03907	0.09946	0.02541	**0.00085**	**0.01709**	**0.03198**
NH	10.417±2.937	0.66892±0.22443	0.86879±0.07129	0.25678	0.00000	**0.00024**	**0.00305**	0.71533
SC	9.583±3.502	0.46063±0.32751	0.85015±0.06778	0.46771	0.00000	**0.00024**	**0.00525**	0.25928
TK	6.500±1.508	0.79762±0.25452	0.85989±0.06130	0.05405	0.24144	**0.00024**	**0.00232**	**0.02613**
**Yushan Mt.**	5.167±1.586	0.60000±0.28284	0.82778±0.10557	0.44444	0.00293	**0.00171**	**0.00122**	**0.02124**
YS	5.167±1.586	0.60000±0.28284	0.82778±0.10557	0.44444	0.00293	**0.00171**	**0.00122**	**0.02124**

*A*, observed average number of alleles; *H*_o_, observed heterozygosity; *H*_e_. expected heterozygosity; *F*_IS_, deviation from Hardy-Weinberg proportions within subpopulations; IAM, infinite alleles model; TPM, two-phase model; SMM, stepwise mutation model. Significant results for Wilcoxon test are in bold.

### Population genetic structure

Based on the neighbor-joining trees for the organellar and nuclear DNA haplotypes of *J*. *morrisonicola*, *J*. *formosana*, and *J*. *chinensis* var. *tsukuiensis* (S1 Fig), no geographical subdivisions were detected as genetic composition was intermixed between populations. To unravel the relative ages among haplotypes/clades, a minimum spanning network (S2 Fig) based on the mutational changes among haplotypes was reconstructed. All widespread haplotypes across loci were nested in the network as interior nodes, indicating their ancestry. The samova program was used to define and identify groups of genetic uniqueness among the 9 populations of *J*. *morrisonicola* (Table 4). For cpDNA, an assumption with two groups (*K* = 2) displayed the greatest value of *F*_CT_ and maximal variance, although nonsignificant (*F*_CT_ = 0.36698, 36.70%, *P* > 0.05). The groups for *K* = 2 did not match the geographical distributions, with the TK population forming one group and the remaining forming the other group. The SAMOVA of mtDNA sequences also revealed the greatest *F*_CT_ value and a statistically maximal variance between groups at *K* = 2 (*F*_CT_ = 0.32643, 32.64%, *P* < 0.05), whereas without matching any geographical distribution. The pattern for the SAMOVA of nuclear sequences also suggested *K* = 2, with the SS population differentiated from all others. In contrast, the microsatellites revealed a pattern different from the sequence data. In total, 8 groups were identified, with low correspondence to the geographical distribution (Table 4), suggesting recent divergence among populations.

**Table 4:** Fixation indices (*F*_CT_) corresponding to groups of populations inferred by samova for populations of *Juniperus morrisonicola* tested for cpDNA, mtDNA, and nrDNA loci and microsatellites.

Marker	Group	Population groupings	*F*_CT_	Variations among group (%)	*P*
cpDNA	2	(SS, DB, HH, HK, KS, NH, SC, YS)(TK)	0.36698	36.70	0.10850
	**3**	**(SS, DB, HH, HK, NH, YS)(KS, SC)(TK)**	**0.35967**	**35.97**	**0.00489**
	4	(SS, DB, HH, HK, NH)(KS, SC)(TK)(YS)	0.35072	35.07	0.00489
	5	(SS, DB, HH, HK, NH)(KS)(SC)(TK)(YS)	0.34356	34.36	0.00800
	6	(SS, DB, HK, NH)(HH)(KS)(SC)(TK)(YS)	0.34619	34.62	0.00684
	7	(SS, DB, NH)(HH)(HK)(KS)(SC)(TK)(YS)	0.33979	33.98	0.00978
	8	(SS, DB)(HH)(HK)(KS)(NH)(SC)(TK)(YS)	0.33849	33.85	0.01662
mtDNA	**2**	**(SS)(DB, HH, HK, KS, NH, SC, TK, YS)**	**0.32643**	**32.64**	**0.00000**
	3	(SS)(DB, HH, HK, KS, NH, SC, YS)(TK)	0.22746	22.75	0.00000
	4	(SS)(DB)(HH, HK, KS, NH, SC, YS)(TK)	0.16530	16.53	0.00489
	5	(SS)(DB)(HH, KS, NH, SC, YS)(HK)(TK)	0.15994	15.99	0.00684
	6	(SS)(DB)(HH, KS, NH, YS)(HK)(SC)(TK)	0.15578	15.58	0.00196
	7	(SS)(DB)(HH, KS, YS)(HK)(NH)(SC)(TK)	0.14926	14.93	0.00782
	8	(SS)(DB)(HH)(HK)(KS, YS)(NH)(SC)(TK)	0.14460	14.46	0.03812
nrDNA	**2**	**((SS)(DB, HH, HK, KS, NH, SC, TK, YS)**	**0.06083**	**6.08**	**0.00000**
	3	(SS)(DB, HH, HK, NH, SC, TK, YS)(KS)	0.45830	4.58	0.02835
	4	(SS)(DB)(HH, HK, NH, SC, TK, YS)(KS)	0.03963	3.96	0.00000
	5	(SS)(DB)(HH, HK, NH, SC, YS)(KS)(TK)	0.03904	3.90	0.00000
	6	(SS)(DB)(HH, HK, SC)(NH, YS)(KS)(TK)	0.04089	4.09	0.00000
	7	(SS)(DB)(HH, HK, SC)(KS)(NH)(TK)(YS)	0.030871	3.87	0.00000
	8	(SS)(HH)(HK, SC)(KS)(TK)(YS)	0.03044	3.04	0.06158
Microsatellites	2	(SS, DB, HH, KS, NH, SC, YS)(HK, TK)	0.01334	1.33	0.00000
	3	(SS)(SS, DB, HH, KS, NH, SC, YS)(HK, TK)	0.01613	1.61	0.00000
	4	(SS, HH, KS, SC, YS)(DB)(HK, TK)(NH)	0.08681	8.68	0.00000
	5	(SS)(DB, NH, SC, YS)(SC)(YS)	0.02044	2.04	0.00000
	6	(SS, SC, YS)(DB)(HH, HK)(KS)(NH)(TK)	0.09103	9.10	0.00489
	7	(SS)(DB)(SC, YS)(HH, HK)(KS)(NH)(TK)	0.09090	9.09	0.01662
	**8**	**(SS)(DB)(HH, HK)(KS)(NH)(SC)(TK)(YS)**	**0.09288**	**9.288**	**0.00000**

Significant results are labeled in bold.

**Fig 4:**
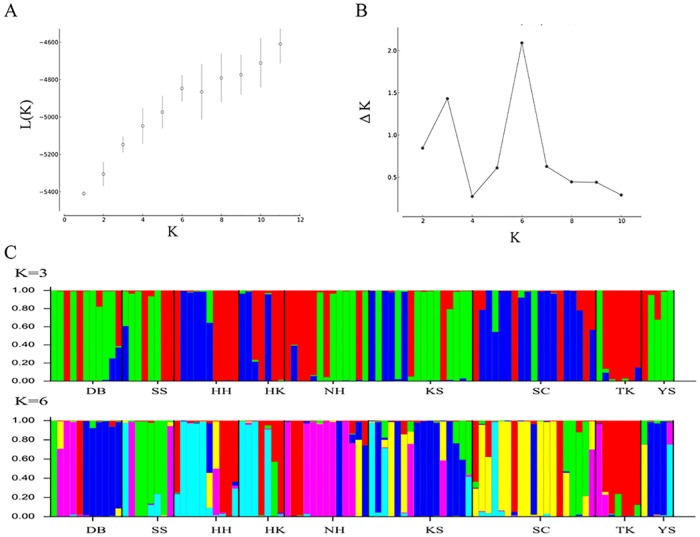
Bayesian inference of the number of clusters (*K*) of *Juniperus morrisonicola* based on microsatellites. (A) The log likelihood for each K. (B) The scatter plots of ΔK. The ΔK is based on the rate of change of ln *P*(*X*/*K*) between successive K values. (C) Six clusters (*K* = 6) were detected based on structure analyses with highest Δ*K* value.

Microsatellite data were analyzed to examine the genetic structure using structure program. As Δ*K* values peaked at *K* = 3 (Δ*K* = 1.4316) and *K* = 6 (Δ*K* = 2.0916), we assigned the proportion of each individual in each population among 3 and 6 clusters, respectively (Fig 4). At K = 3, red component was predominant in the Central Mountain Range (CMR), blue component exclusively occurred in the center of CMR (HH, HK, SC), while green component mostly occurred in peripheral regions of CMR (NH, TK, KS), Sheishan Mountain Range, and Yushan (Fig 4A). At K = 6, although most populations displayed different predominant genetic components, no clear geographical subdivisions were detected among mountain ranges (Fig 4B). Altogether, low levels of genetic differentiation were found across the populations.

### Gene flow

Although the three mountain ranges in Taiwan act as geographical barriers for the migration of *J*. *morrisonicola*, no clear genetic differentiation was detected among different mountain ranges, indicating that some cohesive forces might have existed, e.g. gene flow. To examine the effects of historical gene flow on the populations of *J*. *morrisonicola*, IMa analyses based on all datasets were conducted. The marginal posterior density distributions representing estimated parameters of effective population sizes (*θ*) and the effective number of migrants per generation (*m*) for the comparisons between mountains were indicated (S3 Fig). The effective population size of the Central Mountain (*θ*_Central_) was larger than that of the Sheishan and the Yushan Mountains. For the historical gene flow, Yushan Mountains to Central Mountains (4.05) was highest, followed by from Yushan to Sheishan (3.64), from Central to Sheishan (3.54), and from Sheishan to Central (1.27) (S3 Fig). Results from IMa analyses revealed that non-zero migration may have occurred among the three mountain ranges, with Yushan serving as a source of the gene flow, Central Mountains as a bridge, and Sheishan likely as a sink, altogether implying a northward migration.

### Population demography

In this study, Tajima’s *D* statistic yielded negative values for all loci, except for *Pgi*. Nevertheless, only *Chs*, *Maldehy*, and *Myb* loci showed significant results. At the population level, values for most populations were negative but nonsignificant, with an exception in populations of the CMR at the *Chs* and *Maldehy* loci (Table 2).

**Fig 5:**
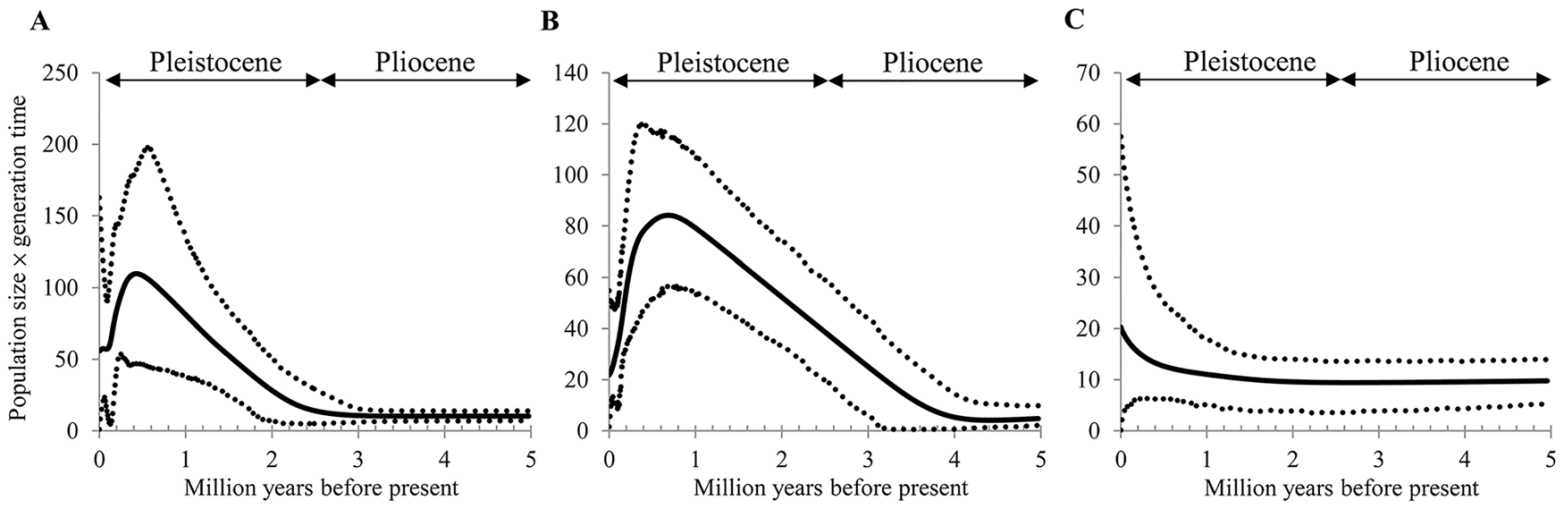
Bayesian skyline plots for *Juniperus morrisonicola*. Bayesian skyline plots for the effective population size fluctuation through time based on nucleotide sequences. The bold line represents the mean estimation, while the area between dashed lines represents the 95% confidence interval. (A) All populations, (B) northern populations, and (C) southern populations.

The Bayesian skyline plots (BSP) were used to uncover the demographic histories of *J*. *morrisonicola* (Fig 5). Till 3 million year ago (late Pliocene), the ancestral population of *J*. *morrisonicola* had maintained a constant population size for a certain time. Following such equilibrium, the ancestral population must have experienced a long-term expansion and reached to a maximum around 0.5 Ma. Subsequently, the population shrunk quickly during the last glacial maximum (around 0.02 Ma), corresponding to the alpine glacier development in Taiwan [10–11] (Fig 5A). As glacier development might be associated with the population dynamics of *J*. *morrisonicola*, we divided the conifer into two groups, i.e., the northern group with populations northern to SC, where the southern limit of alpine glacier occurred, vs. southern group with the remaining populations of TK and KS. Interestingly, demographic analyses revealed that the northern populations may have experienced demographic shrinkage after 1 Ma, whereas southern population expanded simultaneously, implying that the southern populations might have originated from some retreated populations as the extremely global cooling occurred during late Pleistocene (Fig 5B and 5C). On the other hand, microsatellite data also revealed similar scenario, as shown by nucleotide sequences (S4 Fig). To test the scenarios suggested by BSP, approximate Bayesian computation (ABC) with the program DIYABC was conducted. Of the 4 plausible scenarios (Fig 2), the scenario 2, which revealed an early shrinkage of the northern populations followed by the split of southern populations, was best supported by both sequence and microsatellite data (S5 Fig). ABC and BSP analyses contradicted to each other in recovering a recent expansion of the southern population, whereas agreeing in inferring a history of the demographic split of the *Juniperus* populations.

Furthermore, in order to assess the impacts of climate changes on the effective population size of the species, we re-analyzed 14 other plant species in Taiwan based on previously published DNA sequence data. A long history of constant population size of *L*. *formosana* and *C*. *taitungensis* that are distributed at lower elevations of the island, followed by subsequent a slow population growth, was uncovered. The results also revealed that most of these 14 species in Taiwan have undergone recent demographic expansion, except for *A*. *kawakamii* and *C*. *glauca* (S6 Fig).

## Discussion

### Loss of genetic diversity from *Juniperus* populations under climate changes

As a natural geodynamic trend, worsening by the anthropogenic activities, global climate change affecting Earth inevitably influences the dynamics of various organisms and subsequently threatens human survival. Accelerating global warming is expected to affect the migration and distribution of species. In particular, the ability of plants to respond to climate change may be highly variable among species due to the great variation in their life histories and dispersal abilities [53], resulting in different patterns of demographic fluctuations. On an island, where biomes with different vegetation formations are distributed along altitudes, species at different elevations tend to behave differently in response to global warming. For instance, plants growing at lower elevations are likely forced to migrate upward, as Jump et al. [54] found that the upper altitudinal limits of mountain plant distributions have risen by ca 3.6 m yr^-1^ in Taiwan during the last century. Compared to the habitats of middle elevations in Taiwan that are mostly connected to each other along a mountain range, high mountains separated by valleys in contrast represent ecological islands. Today, given hindered dispersal across mountain ranges, a plant exclusively residing the alpine peaks, e.g., *J*. *morrisonicola*, would be greatly constrained by limited area at summits. The differences in altitude gradient among populations of *J*. *morrisonicola* varied from ca. 3200 m to 3900 m, unfortunately there are no habitable area at the higher elevations along its current distributional ranges for populations of *J*. *morrisonicola* to grow and persist in Taiwan. In this study, average genetic diversity of populations was negatively correlated with altitude (Fig 2). Generally, decreasing temperatures associated with increasing altitudes cause a decline in the resource availability. Loss of genetic polymorphisms from shrinking populations likely occurred at the upper edge of the altitudinal range. Although the sample size of this study that yielded relatively higher variances across genetic markers may not be able to provide an accurate estimate of the effective population size, this result implied that the altitudinal gradient acted as a major driver of population genetic diversity in this alpine species.

Under the present climate changes, alpine plants need sufficient genetic diversity for survival; upward/northward migration and colonization are often followed by genetic bottlenecks and a decrease in population genetic diversity. Compared with the nucleotide diversity of alpine *R*. *pseudochrysanthum* in Taiwan (cpDNA, *π* = 0.011, and nrDNA, *π* = 0.011) [50], as well as *P*. *luchuensis* complex in East Asia (cpDNA, *π* = 0.050–0.070, mtDNA, *π* = 0.016–0.033) [45], much lower levels of genetic variation were detected in *J*. *morrisonicola* based on organellar and nuclear markers (Table 2). We also compared the nucleotide diversity of *J*. *morrisonicola* with that of its congeneric species at the same nuclear loci. Interestingly, the levels of genetic diversity of *J*. *morrisonicola* (0.00130–0.01283, Table 2) were higher than those of *J*. *przewalskii* at the *Chs*, *Maldehy*, *Myb* and *Pgi* loci [55]. Nevertheless, a similar pattern in the overall nucleotide diversities across nuclear loci was shared by *Juniperus*, with low-to-moderate levels of nucleotide diversity in species, except for *Needly* and *Pgi* loci in this study [55–57]. Besides, for microsatellites, all populations had slightly lower levels of observed heterozygosity (*H*_o_ = 0.449–0.803) than *J*. *thurifera*, an endemic tree in the western Mediterranean basin (*H*_o_ = 0.773–0.822) [58]. In addition, bottleneck events were detected in all populations of *J*. *morrisonicola* (Table 3), suggesting that loss in genetic diversity was likely correlated with the range reduction of *J*. *morrisonicola*, an observation also agreeing with other empirical studies [59–60]. The effects of genetic drift causing loss of genetic polymorphisms are evident in island-like populations following a gradual increase in mean temperature.

### Demographic fluctuations of *J*. *morrisonicola* under the dramatic climate changes in Pleistocene and Holocene

Global temperature kept falling down during the Pleistocene, and the dramatic mid-Pleistocene climate transition (MPT) at 0.9 Ma led to ice ages with 100,000-year cycles [61]. MPT initiates the development of alpine glaciers in subtropical regions, such as the four major glaciations in Qinghai-Tibet Plateau [62]. If glaciers occur in high mountains of Taiwan after MPT, not only distribution ranges but population growth of alpine conifers would be largely affected. Based on the BSP results, *J*. *morrisonicola* showed population expansion before the onset of MPT, and then quickly shrunk especially during 0.55–0.24 Ma, with a statistical support across the 95% confidence interval. This result suggested that alpine glaciers in Taiwan might have been developed as the climate change during MPT. In Taiwan, glacial remains could be observed at several high mountains some 10,000 years ago, corresponding to the Younger Dryas period [63]. At that time, the average global temperature was 3–4°C lower than the present, resulting in the average snow line of 3300–3500 m in Taiwan island, and the southernmost glacial remain found at Sanchashan [10,12]. As Kuanshan and Takuanshan are southern from Sanchashan, where no glacial remains were detected, low impacts of the alternation of glacial and interglacial periods may imply that these mountains were able to act as a refugium for alpine species. According to the BSP result, the northern populations suffering glacial expansion likely experienced shrinkage in the meantime (Fig 5B). In contrast, tendency of population expansion was detected in the southern populations where no glacial interference occurred, although with a wide confidence interval in the recent geological period (Fig 5C). DIYABC analyses suggested that the initiation of the southern populations occurred via splits from the northern populations that experienced genetic bottlenecks, and that subsequent demographic expansion of the southern populations might have occurred as revealed by the BSP analysis. In addition, although the population sizes in northern Taiwan (Sheishan Mountain) are often larger than those in south, we found that these populations of *J*. *morrisonicola* with lower levels of genetic polymorphisms, especially those inhabiting mountaintops, faced a higher risk of extinction. For other alpine *R*. *pseudochrysanthum* complex in Taiwan, the northern populations had lower level of genetic variations than southern ones [64]. Compared with the other sympatric alpine plants, *J*. *morrisonicola* showed lower fecundity of seedling in the field [65], suggesting that stochastic environmental fluctuation would trigger the decline of populations. Ecological survey and genetic analyses of populations of this long-lived gymnosperm species uncovered such a history of population shrinking, indicating a high risk of extinction of these alpine species. These results suggested that alpine glacier forced northern populations moving downward and experienced fluctuation of distribution, resulting in reduction of genetic diversity; southern populations were formed via splits from the northern populations as the downward migration of mid-elevation forest released a novel niche, also enabling the populations to escape from the alpine glaciers. On the other hand, BSP analyses revealed that most species of lower elevations or with a wide altitudinal range in Taiwan have undergone recent demographic expansion, except for *C*. *glauca*, a species distributed on lowland mountain peaks (S6 Fig). Interestingly, demographic shrinking was also detected in *A*. *kawakamii*, a dominant species of subalpine forests at elevations lower than the *Juniperus* forests. Taken together, global warming may substantially enable many plants at low and mid-elevations to thrive, but hinder those at high elevations [66]. Accordingly, in contrast to the well-documented postglacial demographic expansion of most lowland species [9], species in alpine habitats would tend to shrink in size and distributional span.

### Vulnerability and lack of genetic differentiation among fragmented *Juniperus* populations

Here, we showed that *J*. *morrisonicola* might be threatened by the historical alpine glaciers. The surviving populations might have been shaped by local adaptation, range shifts, and other stochastic factors [67–69]. Of these, range reduction is likely to cause the loss of genetic diversity [70], which in turn may severely limit the species’ responses to environmental change. Following the subsequent deglaciation, analogous to tree line shifts to the poles [71], elevating global temperatures triggered upward shifts along the tree line [72–74]. In Taiwan, Jump et al. [54] found that the upper altitudinal limits of mountain plant distributions have risen by ca 3.6 m yr^-1^ during the last century, in parallel with rising temperatures in the region. Responding to the increasing temperatures, migration of alpine species at local peaks would be largely constrained, inevitably resulting in high rates of extinction [75]. A previous study based on expressed sequence tag-simple sequence repeats (EST-SSR) found that postglacial climatic changes had significant impacts on population fluctuations and isolation in the *R*. *pseudochrysanthum* complex, and that higher-elevation populations had lower effective population sizes than lower-elevation ones, and northern populations also had lower level of genetic diversity than southern ones [64]. If the geographical isolation in *J*. *morrisonicola* was long enough, low levels of genetic diversity and high levels of genetic differentiation among mountain ranges would be expected, due to small, isolated and fragmented patches of *J*. *morrisonicola*.

Rapid climate change may also act as a potent agent of natural selection within populations, which in combination with habitat fragmentation alters population genetic structure [76]. For alpine plants, including *J*. *morrisonicola*, valleys and high mountain ridges could demarcate the plants’ habitats and might consequently impede gene flow among plant populations and opportunities to colonize new habitats [77]. Unexpectedly, genetic analyses based on organellar and nuclear DNA variations revealed very weak genetic differentiation of populations among mountain ranges and failed to support population/mountain range monophyly (S1 Fig). Here, since the occurrence of the last glacial retreat about 10,000 years ago, given a generation time of 50 years for *J*. *morrisonicola*, only about 200 generations have been completed. Given such a short time for isolation between populations, incomplete lineage sorting would lead to maintenance of ancestral polymorphisms within populations [45], explaining the low levels of genetic differentiation at most markers and paraphyly of populations.

On the other hand, the IMa analyses revealed non-zero, historical migration among mountain ranges (S3 Fig). Here, compared with the gene flow among populations of the Sheishan, we found that genetic exchange tended to be asymmetric between mountain ranges, with higher northward migration rates. This result also supported southern mountains might serve as a refugium during the glacial maxima, and thus more migrants dispersed northward after the alpine glacier retreat. Taken together, genetic analyses revealed frequent gene flow between populations/mountain ranges (Fig 4, and S3 Fig), a pattern also detected in *J*. *communis* [78] and *J*. *excelsa* [79]. Today, over an interglacial period, populations of *J*. *morrisonicola* in different mountain ranges grow in isolated and fragmented patches. These results suggested that frequent gene flow might have occurred during the glacial maxima, as the alpine populations shifted downward/southward and connected to each other. As recent gene flow was likely hindered among island-like populations, the trend showing decreasing nucleotide diversity along with increasing altitudes implied population shrinkage in this alpine species. A similar scenario was also found for *Rhododendron pseudochrysanthum*, another alpine plant in Taiwan [80].

## Conclusions

In this study, we found that genetic diversity of populations in *J*. *morrisonicola* decreases with increasing altitudinal gradients. The northern populations at high latitudes were less genetically polymorphic at nuclear and organellar markers than southern ones, suggesting that the development of alpine glaciers had likely led to the loss of genetic polymorphisms. In addition to the effects of incomplete lineage sorting, high rates of historical gene flow via pollen and seeds could also have resulted in the low levels of genetic differentiation among *Juniperus* populations. In contrast, microsatellites supported some levels of genetic differentiation across mountain ranges. Our analyses revealed a scenario of the initial colonization during the early Pleistocene, followed by steady population growth until the approaching of the ice age, which brought the alternation of glacial and interglacial periods and turned the populations into a rapid demographic contraction. In contrast, most other species at lower elevations of Taiwan experienced recent demographic expansion. Apparently, these climate changes had significant impacts on the demography of *J*. *morrisonicola*, as well as other alpine conifers, such as *Abies*, on this island. Nevertheless, these populations should not be considered as a dead-end destined for deterministic extinction and perhaps as a focus of conservation efforts.

## Supporting Information

S1 Fig Neighbor-joining trees of *Juniperus morrisonicola*. The phylogeny based on haplotype sequences were reconstructed with MEGA 5. Bootstrap values are indicated at nodes. (A) *trn*S*-trnG*; (B) *trnT*-*trnL*; (C) *coxI*; (D) *coxIII*; (E) *Chs*; (F) *Maldehy*; (G) *Myb*; (H) *Needly*; (I) *Pgi*.
(PDF)

S2 Fig Minimum spanning network of of *Juniperus morrisonicola*. (A) *trnS-trnG*; (B) *trnT*-*trnL*; (C) *coxI*; (D) *coxIII*; (E) *Chs*; (F) *Maldehy*; (G) *Myb*; (H) *Needly*; (I) *Pgi*.
(PDF)

S3 Fig Illustrations of the marginal posterior densities distributions representing estimated. (A) effective population sizes (θ), and (B) the effective number of migrants per generation (*m*) for the comparison between mountains. (**C**) Diagram of migration numbers and effective population sizes for *Juniperus morrisonicola*. The thickness of the lines corresponds to the fraction of migrating individuals per generation (*M*), and the area of the circle corresponds to the effective population sizes.
(PDF)

S4 Fig Bayesian skyline plots for the effective population size fluctuation through time based on microsatellites. The bold line represents the mean estimation, while the area between dashed lines represents the 95% confidence interval. (A) All populations, (B) northern populations, and (C) southern populations.
(PDF)

S5 Fig Logistic regression of posterior probability of four plausible scenarios. X-axis presents number of simulated datasets. Y-axis presents the proportion of supported scenarios. Scenarios are denoted by different colors. (A) Sequence data, (B) microsatellite data.
(PDF)

S6 Fig Bayesian skyline plots for 14 plant species in Taiwan. The fluctuation in effective population size over time for 14 plant species (a–n) based on previously published DNA sequences (S3 Table). Black lines represent mean estimations; area between dashed-lines represents 95% confidence intervals.
(PDF)

S1 Table The forward (F) and reverse (R) sequences of primers for each gene used in this study. (PDF)

S2 Table The forward (F) and reverse (R) sequences, repeat motif, allele size (bp), and annealing temperature (Tm) of microsatellites are indicated. (PDF)

S3 Table List of the sequences of 14 plant species used in beast skyline analyses. (PDF)

S4 Table Prior distributions and summary statistics used in DIYABC analyses. (PDF)

S5 Table Haplotype distribution of 9 sequence markers. (PDF)
