# Supplementary material for: Multilocus Analyses Reveal Postglacial Demographic Shrinkage of Juniperus morrisonicola (Cupressaceae), a Dominant Alpine Species in Taiwan
Source: PLoS One. 2016 Aug 25;11(8):e0161713. doi: 10.1371/journal.pone.0161713 (PMC4999204; doi:10.1371/journal.pone.0161713)
Supplement: S1 Fig — The phylogeny based on haplotype sequences were reconstructed with MEGA 5. Bootstrap values are indicated at nodes. (A) trnS-trnG; (B) trnT-trnL; (C) coxI; (D) coxIII; (E) Chs; (F) Maldehy; (G) Myb; (H) Needly; (I) Pgi. (PDF) [file pone.0161713.s001.pdf]

S1 Figure

A *trnS-trnG*

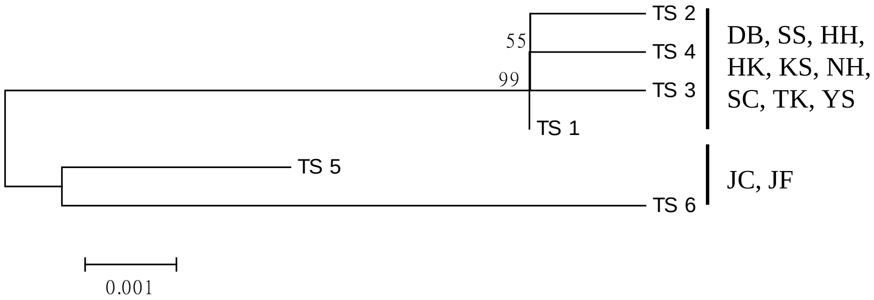

B *trnT-trnL*

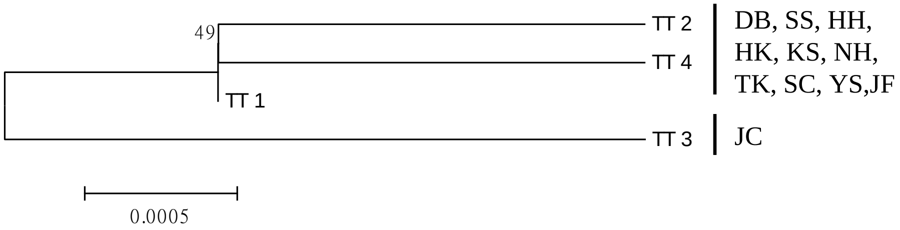

C *coxI*

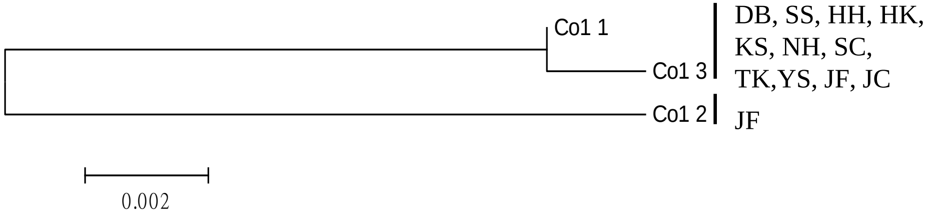

D *coxIII*

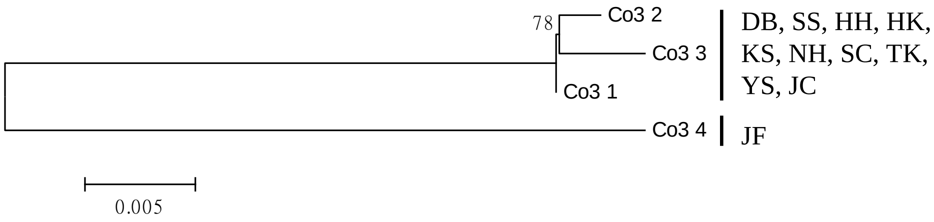

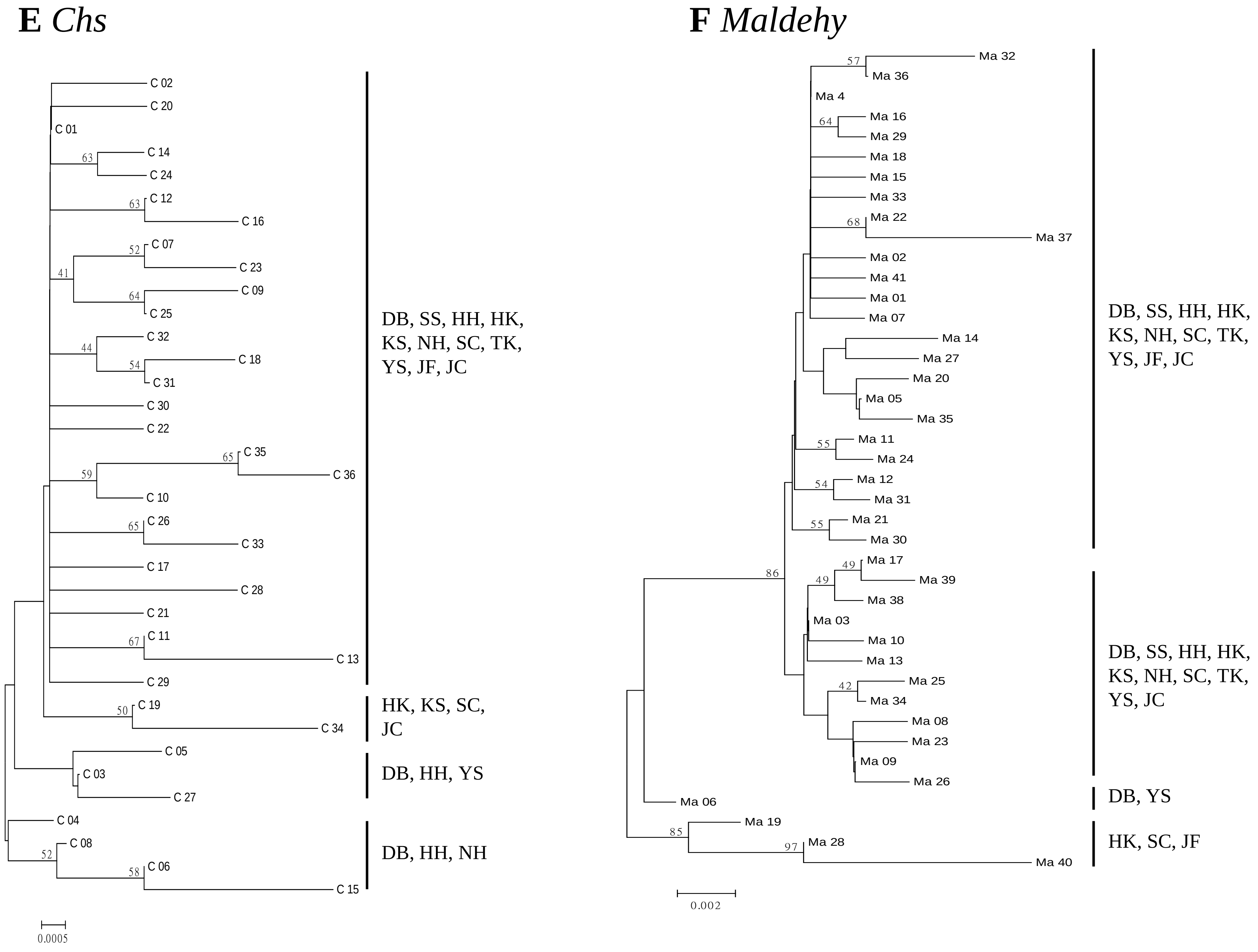
E Chs
C 02
C 20
C 01
C 14
C 24
C 12
C 16
C 07
C 23
C 09
C 25
C 32
C 18
C 31
C 30
C 22
C 35
C 36
C 10
C 26
C 33
C 17
C 28
C 21
C 11
C 13
C 29
C 19
C 34
C 05
C 03
C 27
C 04
C 08
C 06
C 15
DB, SS, HH, HK, KS, NH, SC, TK, YS, JF, JC
HK, KS, SC, JC
DB, HH, YS
DB, HH, NH
0.0005
F Maldehy
Ma 32
Ma 36
Ma 4
Ma 16
Ma 29
Ma 18
Ma 15
Ma 33
Ma 22
Ma 37
Ma 02
Ma 41
Ma 01
Ma 07
Ma 14
Ma 27
Ma 20
Ma 05
Ma 35
Ma 11
Ma 24
Ma 12
Ma 31
Ma 21
Ma 30
Ma 17
Ma 39
Ma 38
Ma 03
Ma 10
Ma 13
Ma 25
Ma 34
Ma 08
Ma 23
Ma 09
Ma 26
Ma 06
Ma 19
Ma 28
Ma 40
DB, SS, HH, HK, KS, NH, SC, TK, YS, JF, JC
DB, SS, HH, HK, KS, NH, SC, TK, YS, JC
DB, YS
HK, SC, JF
0.002

G *Myb*

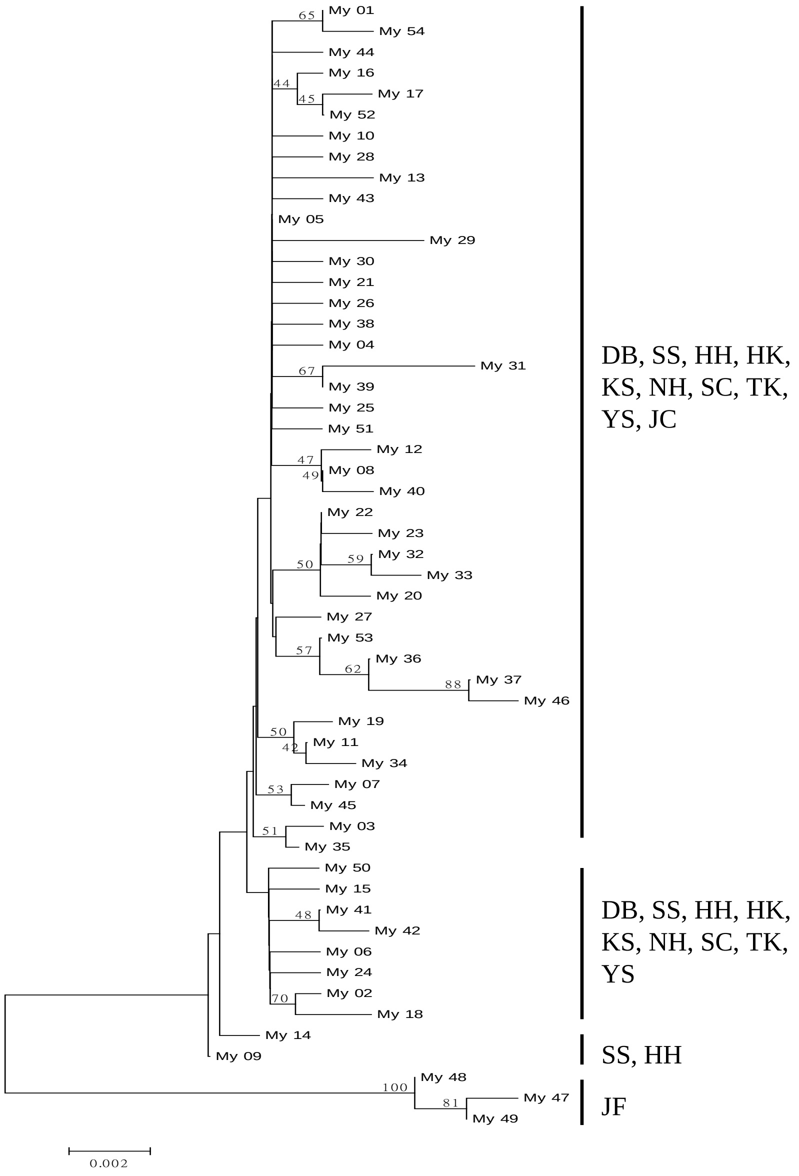

H *Needly*

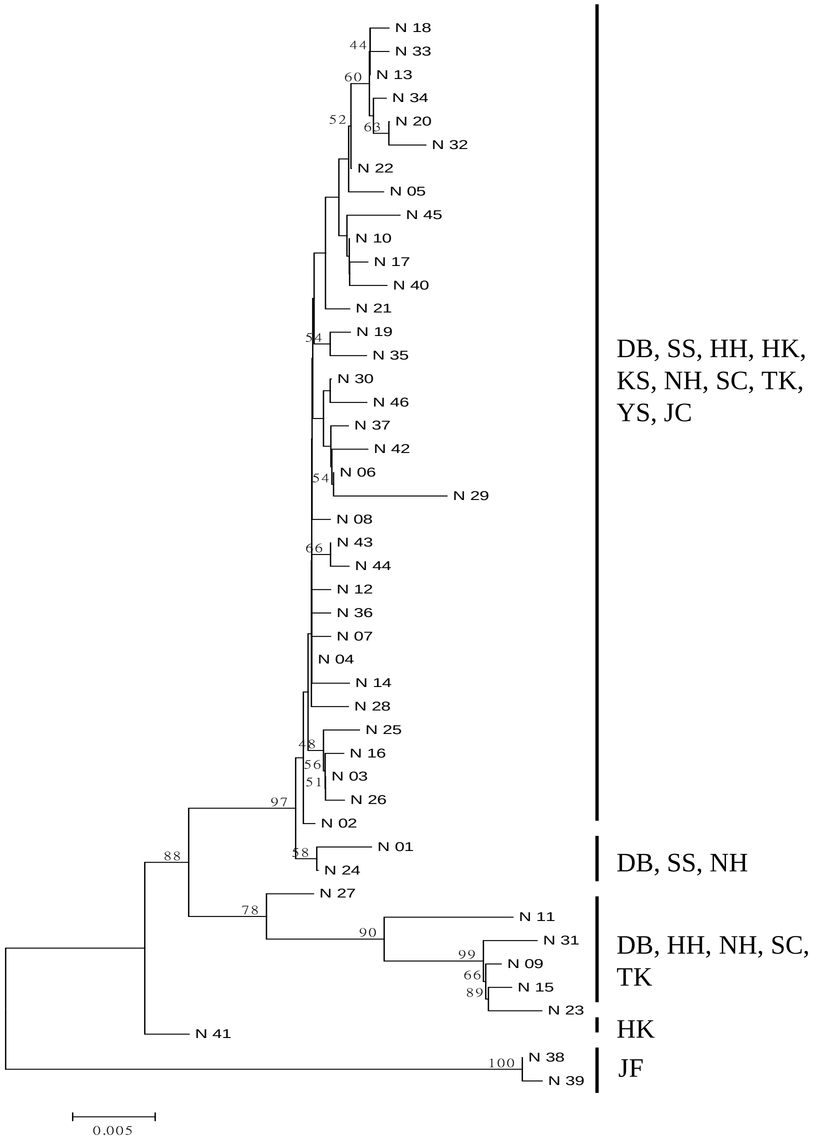

I *Pgi*

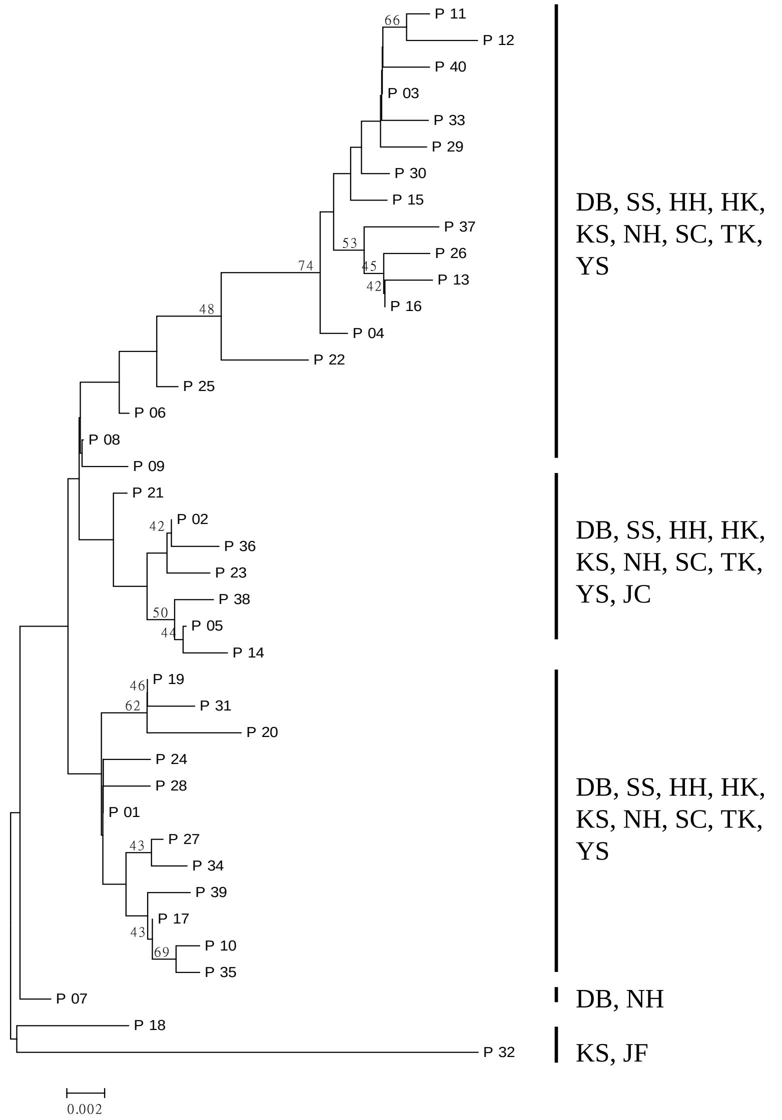
P 11
66
P 12
P 40
P 03
P 33
P 29
P 30
P 15
P 37
53
P 26
74
45
P 13
42
P 16
48
P 04
P 22
P 25
P 06
P 08
P 09
P 21
P 02
42
P 36
P 23
P 38
50
P 05
44
P 14
P 19
46
62
P 31
P 20
P 24
P 28
P 01
P 27
43
P 34
P 39
P 17
43
P 10
69
P 35
P 07
P 18
P 32
0.002
DB, SS, HH, HK,
KS, NH, SC, TK,
YS
DB, SS, HH, HK,
KS, NH, SC, TK,
YS, JC
DB, SS, HH, HK,
KS, NH, SC, TK,
YS
DB, NH
KS, JF
