# Supplementary figures and images for: Multilocus Analyses Reveal Postglacial Demographic Shrinkage of Juniperus morrisonicola (Cupressaceae), a Dominant Alpine Species in Taiwan
Source: PLoS One. 2016 Aug 25;11(8):e0161713. doi: 10.1371/journal.pone.0161713 (PMC4999204; doi:10.1371/journal.pone.0161713)

S2 Figure

**A** *trnS-trnG*

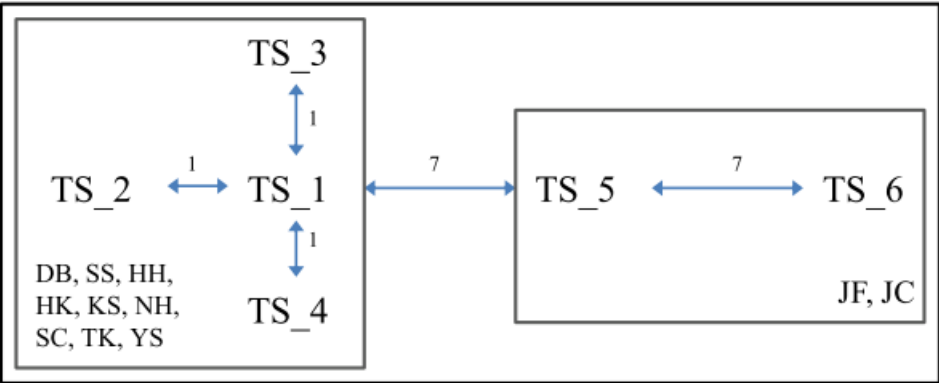

**B** *trnT-trnL*

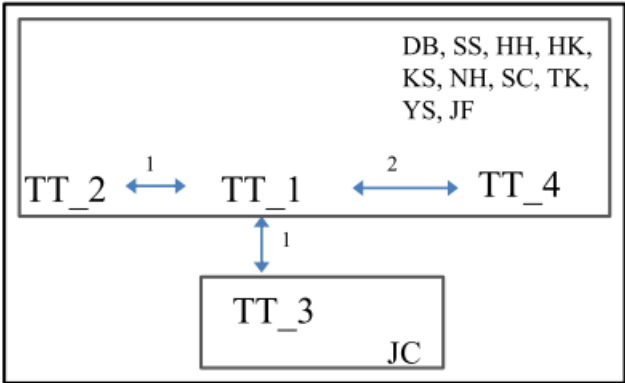

**C** *coxI*

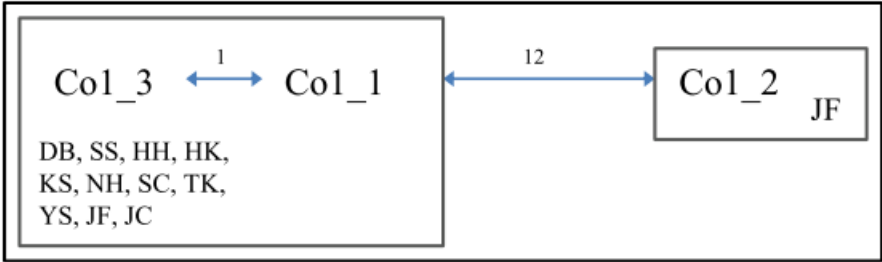

**D** *coxIII*

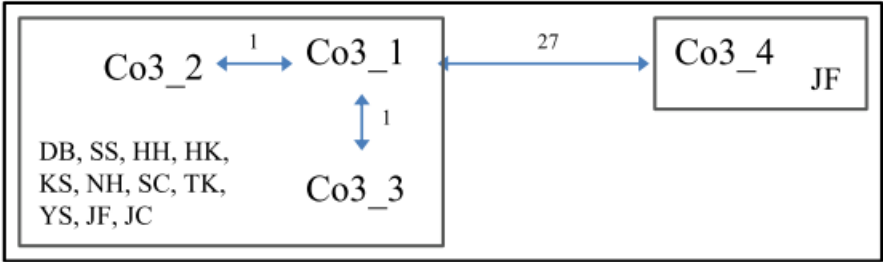

## E Chs

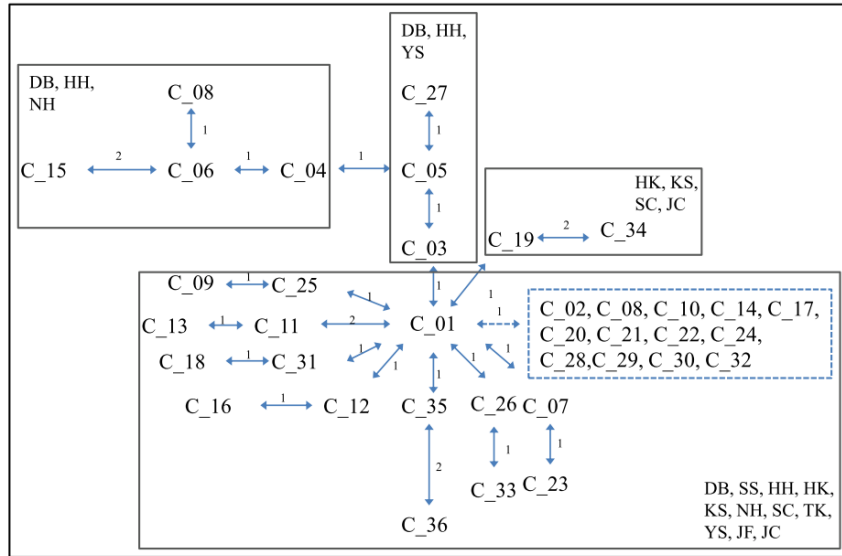

## F Maldehy

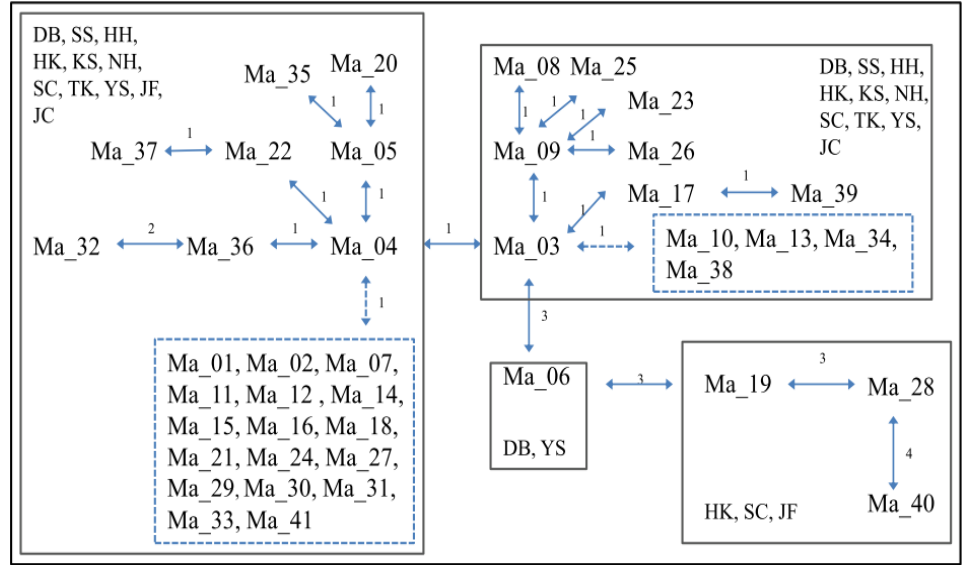

## G Myb

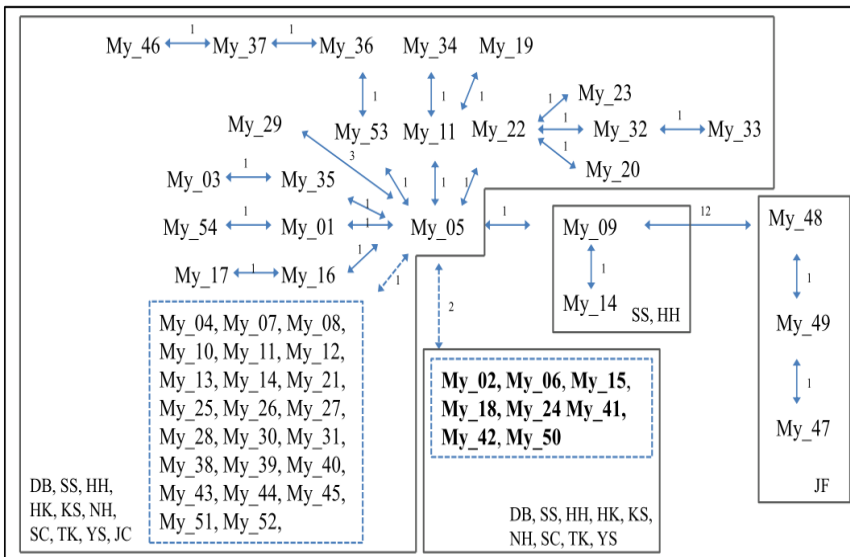

## H Needly

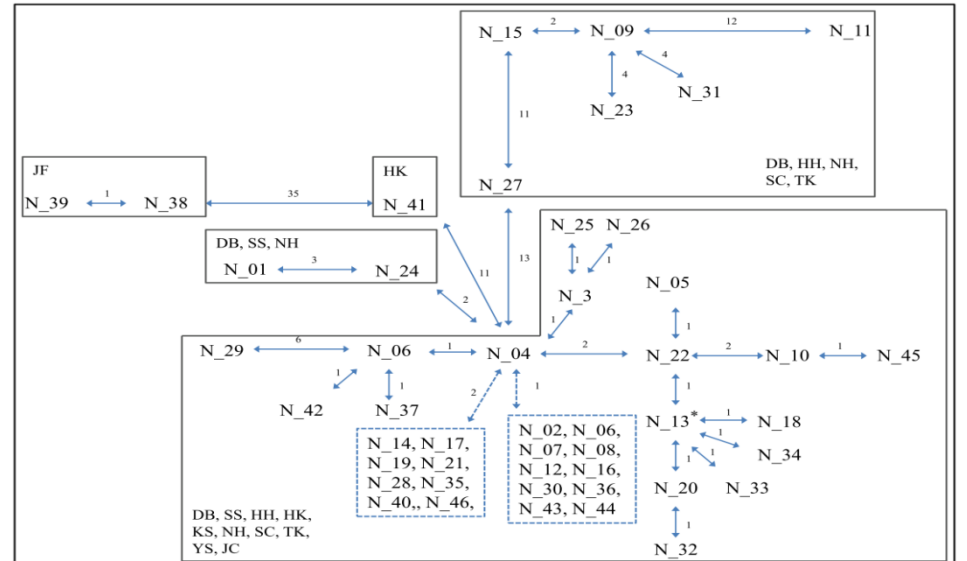

Supplement: S2 Fig — (A) trnS-trnG; (B) trnT-trnL; (C) coxI; (D) coxIII; (E) Chs; (F) Maldehy; (G) Myb; (H) Needly; (I) Pgi. (PDF) [file pone.0161713.s002.pdf]

# S3 Figure

## A

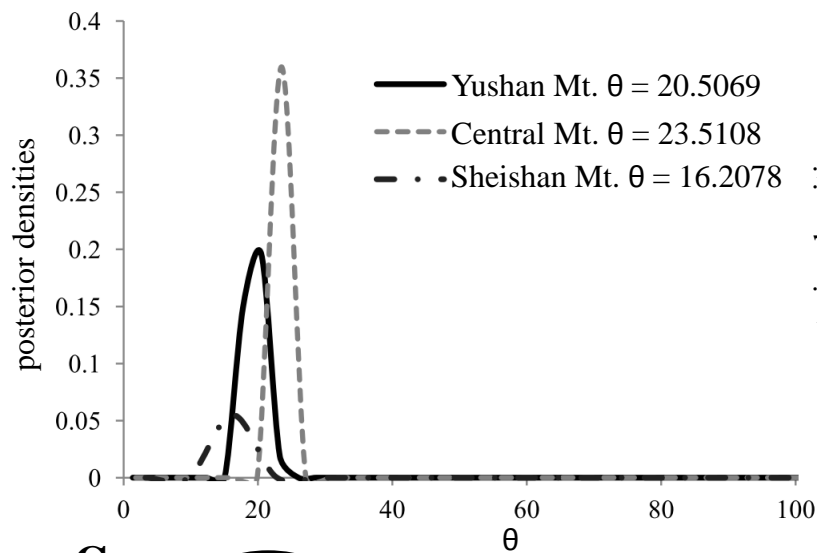

## B

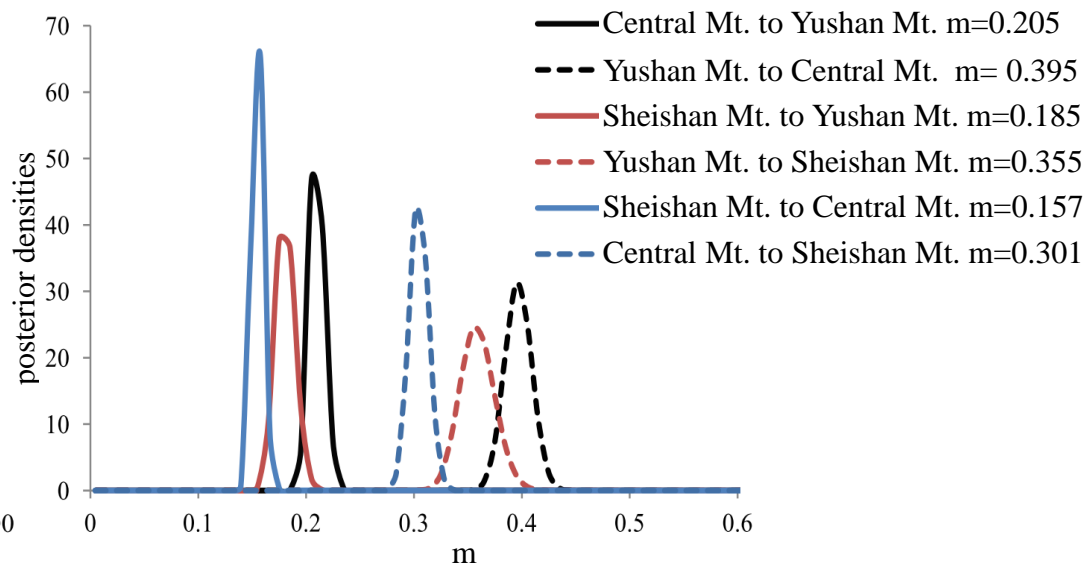

## C

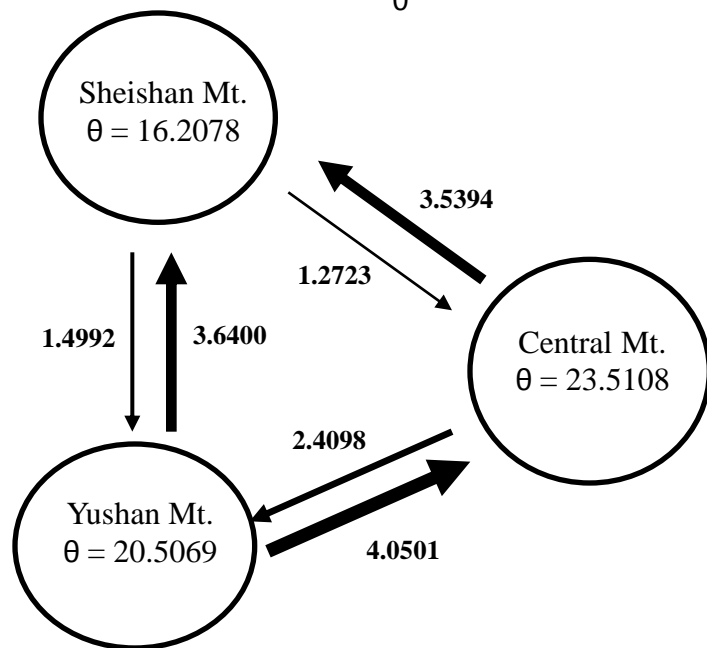

Supplement: S3 Fig — (A) effective population sizes (θ), and (B) the effective number of migrants per generation (m) for the comparison between mountains. (C) Diagram of migration numbers and effective population sizes for Juniperus morrisonicola. The thickness of the lines corresponds to the fraction of migrating individuals per generation (M), and the area of the circle corresponds to the effective population sizes. (PDF) [file pone.0161713.s003.pdf]

S4 Figure

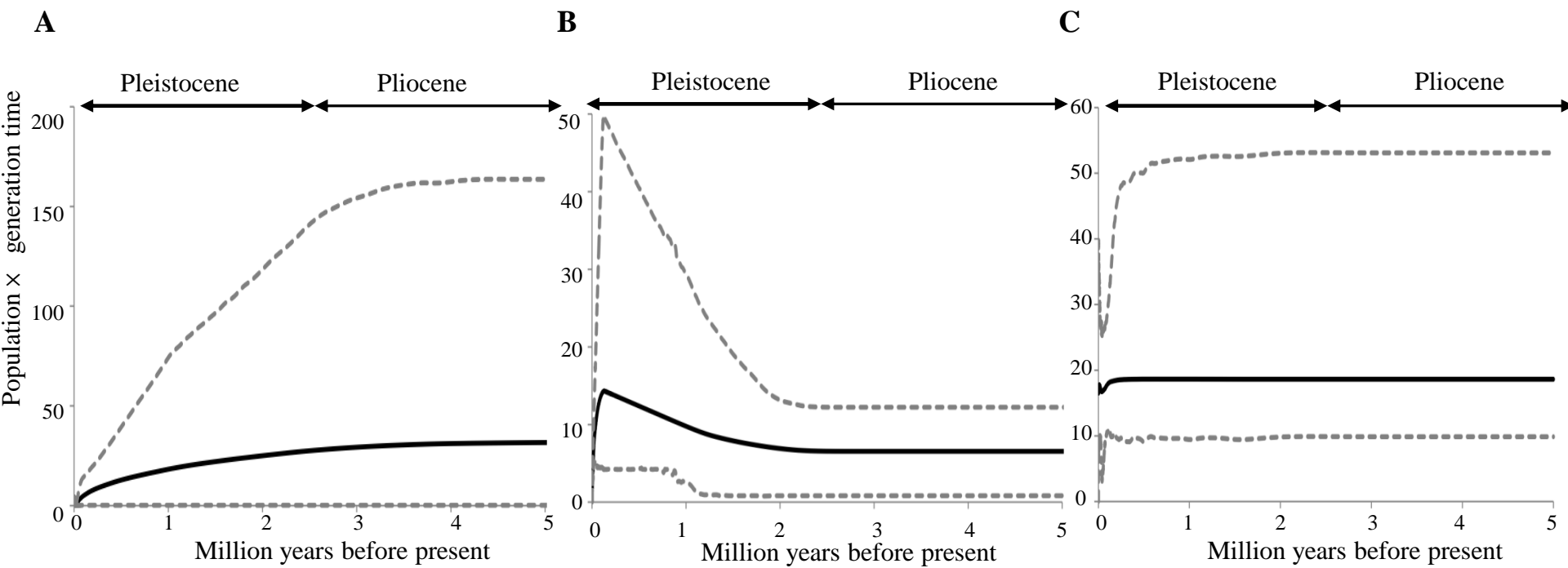

Supplement: S4 Fig — The bold line represents the mean estimation, while the area between dashed lines represents the 95% confidence interval. (A) All populations, (B) northern populations, and (C) southern populations. (PDF) [file pone.0161713.s004.pdf]

## S5 Figure

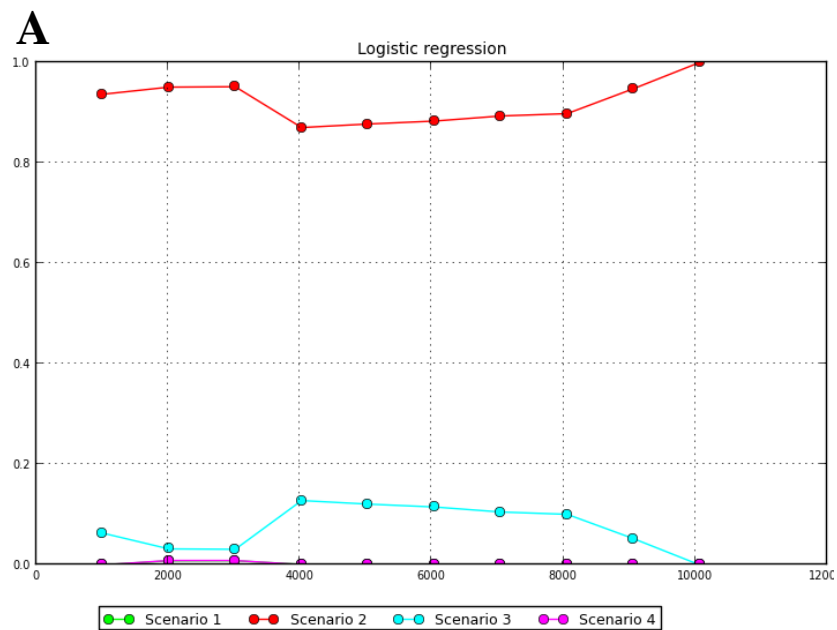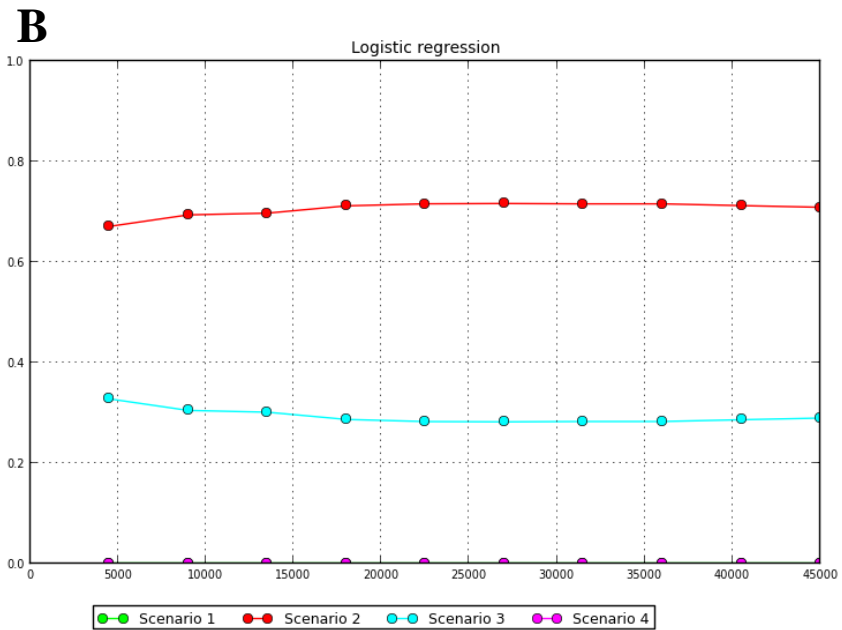

Supplement: S5 Fig — X-axis presents number of simulated datasets. Y-axis presents the proportion of supported scenarios. Scenarios are denoted by different colors. (A) Sequence data, (B) microsatellite data. (PDF) [file pone.0161713.s005.pdf]

# S6 Figure

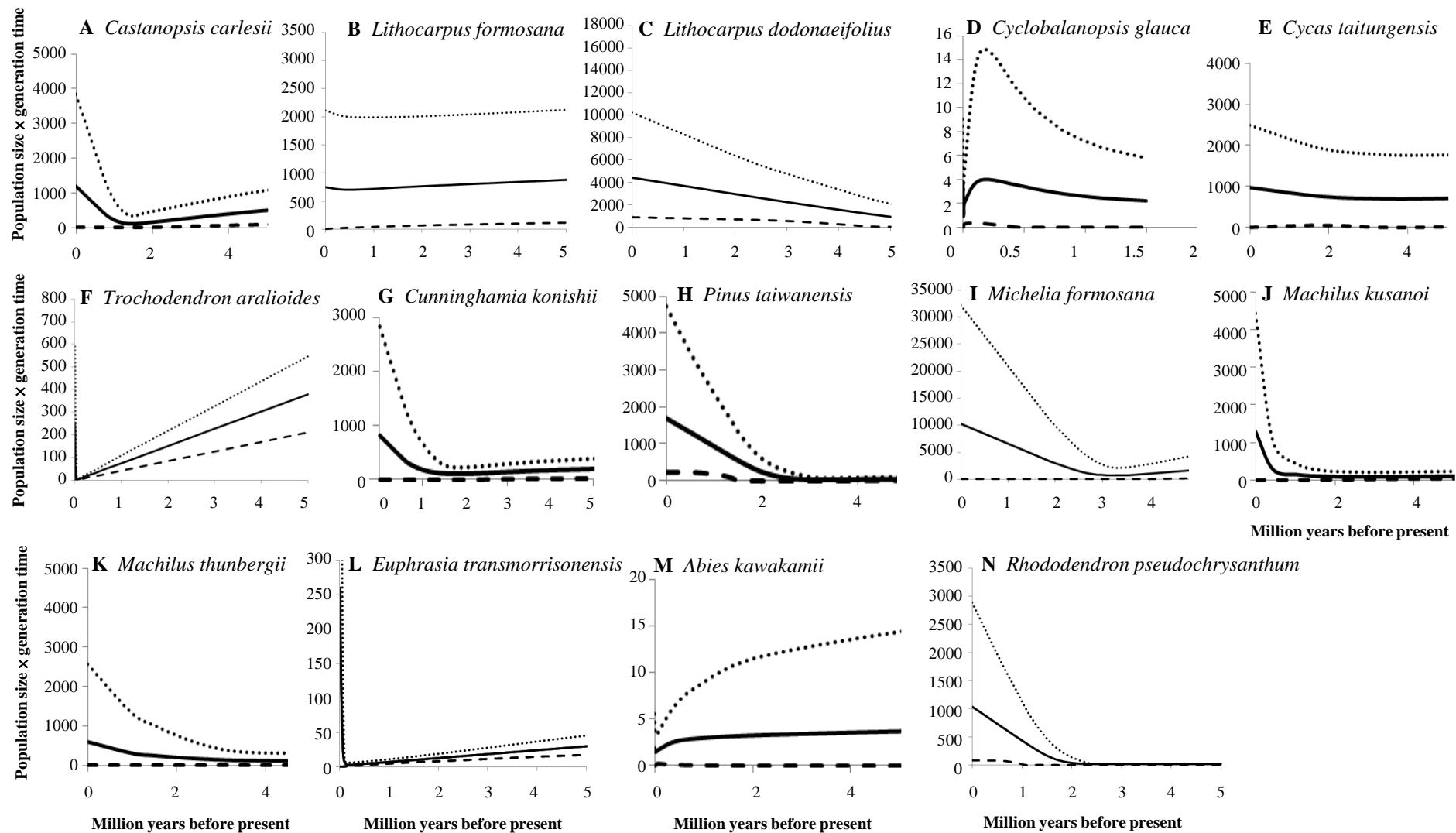

Supplement: S6 Fig — The fluctuation in effective population size over time for 14 plant species (a–n) based on previously published DNA sequences (S3 Table). Black lines represent mean estimations; area between dashed-lines represents 95% confidence intervals. (PDF) [file pone.0161713.s006.pdf]
