# Supplementary material for: Multilocus Analyses Reveal Postglacial Demographic Shrinkage of Juniperus morrisonicola (Cupressaceae), a Dominant Alpine Species in Taiwan
Source: PLoS One. 2016 Aug 25;11(8):e0161713. doi: 10.1371/journal.pone.0161713 (PMC4999204; doi:10.1371/journal.pone.0161713)
Supplement: S1 Table — (PDF) [file pone.0161713.s007.pdf]

S1 Table. The forward (F) and reverse (R) sequences of primers for each gene used in this study.

Gene	Primer sequence (5′–3′)
Chloroplast DNA	
*trn*S-*trn*G	F: GCCGCTTTAGTCCACTCAGC
	R: GAACGAATCACACTTTTACCAC
*trn*T-*trn*L	F: CATTACAAATGCGATGCTCT
	R: ATTTGAACTGGTGACACGAG
Mitochondrial DNA	
*cox*I	F: ACCCTTTTCGATCCTGCTG
	R: CAAGAAATGCATGGGAAAGG
*cox*III	F: ATGTACATGCACTCATTCACG
	R: AACCATGAAACCCAGTTGCT
Nuclear DNA	
Chs	F: GGTCGGTCTGACATTCCATT
	R: CGAGAAGCGTCCAAACATTA
Maldehy	F: CATATCCGTGATTGGGTGCTT
	R: CAGTGGCATCCAGTTTTTCCT
Myb	F: CGAGGATTCCACCATTATCG
	R: TGGAGCAAATGCACTCAATC
Needly	F: ATCTCCAAGGAAAGGGGAGA
	R: GCATGGCGAAATACCTGATT
Pgi	F: GCCCAAAATCCTCTGTGTCT
	R: GACTTCCTGAGCCAATAATG
