# Supplementary material for: Multilocus Analyses Reveal Postglacial Demographic Shrinkage of Juniperus morrisonicola (Cupressaceae), a Dominant Alpine Species in Taiwan
Source: PLoS One. 2016 Aug 25;11(8):e0161713. doi: 10.1371/journal.pone.0161713 (PMC4999204; doi:10.1371/journal.pone.0161713)
Supplement: S2 Table — (PDF) [file pone.0161713.s008.pdf]

S2 Table. The forward (F) and reverse (R) sequences, repeat motif, allele size (bp), and annealing temperature (Tm) of microsatellites are indicated.

Locus	Primer sequences (5′–3′)	Repeat motif	Tm (°C)	Allele size (bp)	Reference
Juni01	F: GCTCAGGCAGAAAGAGAACG	$(AG)_{10}$	51	128–143	Chiu 2010
	R: GGCTTTTGGCTTCGTGTTT				
Juni02	F: AGGACATAATGTGTTAGGGGACA	$(TG)_{12}$	51	230–246	Chiu 2010
	R: GGTCCACACCTATTGTGCTAAAG				
Juni05	F: GGACAAAACCTCATTTGTAATAGTG	$(TG)_{8}$	51	149–176	Chiu 2010
	R: CTAGGTGGACATATCATGCGTA				
Juni06	F: AACTGTGCCGCAATCTCTGT	$(TC)_{10}$	51	112–131	Chiu 2010
	R: GGTGAGGGCTCAAGAGTCAA				
Juni08	F: GAAAAGAAGAGACTGGAGGAAGC	$(AAC)_{5}(GAC)_{2}(AAC)_{2}$	51	196–230	Chiu 2010
	R: AGCAGTTGGAGTTAGTTCGTGAG				
Juni09	F: ACCCGGCAAAAGAAAAGAAG	$(GCAG)_{5}$	52	108–120	Chiu 2010
	R: GTTCCGTGCAGAGGTACCAG				
Juni11	F: ATTGTGGAGCGTTTAATTCGTT	$(AGA)_{8}$	52	139–149	Chiu 2010
	R: GGGAAACAACATCTTTCTGAGC				
Juni14	F: GAGCAGCGGCTAAGAGGAG	$(AAG)_{6}$	52	117–126	Chiu 2010
	R: GGAGCAGCTTGAAACTCACA				
Jp03	F: AGGCCAAATCACTTGAGTATAAC	$(AG)_{12}$	49	130–153	Michalczyk *et al*. 2006
	R: CCTACATGAGTTCCTTCTACACC				
Jp07	F: CATCCTCTTCAGTTAGGGTCC	$(AG)_{5}$	48	179–216	Michalczyk *et al*. 2006
	R: GATTTAGTGGCACCTACATGAG				

Jc32	F: ACATTGCAAATATGGGGTAA	$(AC)_{14}(ATC)_8$	49	163–185	Zhang *et al*. 2008
	R: TTGATGAGTTGTTGAGTTATTAAG				
Jc35	F: TGTGTTTATTCTCCCCATCT	$(CA)_{20}$	49	121–147	Zhang *et al*. 2008
	R: CCCCCAGTTATTCTAAACATT				

Chiu CD: **The population genetic structure of *Juniperus morrisonicola* in Taiwan by using microsatellites DNA markers.** Master thesis. Cheng Kung University, Department of Life Sciences; 2010.

Michalczyk IM, Sebastiani F, Buonamici A, Cremer E, Mengel C, Ziegenhagen B, Vendramin GG: **Characterization of highly polymorphic nuclear microsatellite loci in *Juniperus communis* L.** *Mol Ecol Notes* 2006, **6**:346–348.

Zhang Q, Yang YZ, Wu GL, Zhang DY, Liu JQ: **Isolation and characterization of microsatellite DNA primers in *Juniperus przewalskii* Kom (Cupressaceae)**. Conserv Genet 2008, **9**:767–769.
