# Supplementary material for: Multilocus Analyses Reveal Postglacial Demographic Shrinkage of Juniperus morrisonicola (Cupressaceae), a Dominant Alpine Species in Taiwan
Source: PLoS One. 2016 Aug 25;11(8):e0161713. doi: 10.1371/journal.pone.0161713 (PMC4999204; doi:10.1371/journal.pone.0161713)
Supplement: S3 Table — (PDF) [file pone.0161713.s009.pdf]

**S3 Table**. List of the sequences of 14 plant species used in BEAST Bayesian skyline analyses.

| Species | Loci | Accession numbers | References |
|---|---|---|---|
| *Abies kawakamii* (Hayata) Ito | *gapC* | DQ116577–DQ116586 | Shih *et al*. 2007 |
| | *trnL-trnF* | DQ116573–DQ116575 | |
| *Castanopsis carlesii* (Hemsl.) Hayata | *trnL* | AY714626–AY714646 | Cheng *et al*. 2005 |
| | *trnV-trnM* | AY714620–AY714625 | |
| *Cunninghamia konishii* Hayata | *petG-trnP* | AF549412–AF549415; | Hwang *et al*. 2003 |
| | *trnD-trnT* | AJ274382–AF549417; | |
| | *trnL-trnF* | AF549419, AF549427, AF549429; | |
| | *trnV* | AF549430–AF549440 | |
| *Cycas taitungensis* C. F. Shen *et al.* | ITS | FN397941–FN397954 | Huang *et al*. 2001 |
| | *atpB-rbcL* | FM999764–FM999773 | |
| *Euphrasia transmorrisonensis* Hayata | ITS | AY264991–AY165622, AY513682–AY513689 | Wu *et al*., 2005 |
| | *trnL-trnF* | AY512684–AY512719, AY512727–AY512730, AY512738–AY512774 | |
| *Machilus kusanoi* Hayata | *trnL-trnF* | AY819940–AY819944 | Wu *et al*. 2006 |
| | *trnV-trnM* | AY819945–AY819950 | |
| *Machilus thunbergii* Siebold & Zucc | *trnL-trnF* | AY819940–AY819944 | Wu *et al.* 2006 |
| | *trnV-trnM* | AY819945–AY819950 | |
| *Michelia formosana* (Kaneh.) Masam. & Suzuki | *atpB-rbcL* | AJ270814–AJ270846 | Lu *et al*. 2002 |
| *Pinus luchuensis* Hayata | *atpB-rbcL* | DQ196719–DQ196781 | Chiang *et al*. 2006 |
| | *nad1* | DQ196782–DQ196865 | |

| | | | |
|---|---|---|---|
| *Cyclobalanopsis glauca* (Thunb.) Oerst. | *petG-trnP* | AY091646–AY091649 | Huang *et al*. 2002 |
| | *trnL-trnF* | AY091653–AY091662 | |
| | *trnV-trnM* | AY091650–AY091652 | |
| *Rhododendron pseudochrysanthum* Hayata | ITS | HQ850623 - HQ850657 | Huang *et al*. 2011 |
| | *atpB-rbcL* | HQ850658 - HQ850693 | |
| *Trochodendron aralioides* Siebold & Zucc. | *petA-psbJ* | AY294659–AY294753 | Huang *et al*. 2004 |
| | *petG-trnP* | AY294754–AY294848 | |
| *Lithocarpus formosana* (Hayata) Hayata | *atpB-rbcL* | AJ390792–AJ390810 | Chiang *et al*. 2004 |
| *Lithocarpus dodonaeifolius* (Hayata) Hayata | *atpB-rbcL* | AJ252216–AJ252234, AJ390818–AJ390837 | Chiang *et al*. 2004 |

Cheng YP, Hwang SY, Lin TP: **Potential refugia in Taiwan revealed by the phylogeographical study of *Castanopsis carlesii* Hayata (Fagaceae).** *Mol Ecol* 2005, **14**:2075–2085.

Chiang TY, Hung KH, Hsu TW, Wu WL: **Lineage sorting and phylogeography in *Lithocarpus formosanus* and *L. dodonaeifolius* (Fagaceae) from Taiwan.** *Ann MO Bot Gard* 2004, **91**:207-222.

Chiang YC, Hung KH, Schaal BA, Ge XJ, Hsu TW, Chiang TY: **Contrasting phylogeographical patterns between mainland and island taxa of the *Pinus luchensis* complex.** *Mol Ecol* 2006, **15**:765–779.

Huang S, Chiang YC, Schaal BA, Chou CH, Chiang TY: **Organelle DNA phylogeography of *Cycas taitungensis*, a relict species in Taiwan.** *Mol Ecol* 2001, **10**:2669–2681.

Huang SS, Hwang SY, Lin TP: **Spatial pattern of chloroplast DNA variation of *Cyclobalanopsis glauca* in Taiwan and eastern Asia.** *Mol Ecol* 2002, **11**:2349–2358.

Huang SF, Hwang SY, Wang JC, Lin TP: **Phylogeography of *Trochodendron aralioides* (Trochodendraceae) in Taiwan and its adjacent areas.** *J Biogeogr* 2004, **31**:1251–1259.

Huang CC, Hung KH, Hwang CC, Huang JC, Lin HD, Wang WK, Wu PY, Hsu TW, Chiang TY: **Genetic population structure of the alpine**

**species *Rhododendron pseudochrysanthum* sensu lato (Ericaceae) inferred from chloroplast and nuclear DNA.** *BMC Evol Biol* 2011, **11**:108.

Hwang SY, Lin TP, Ma CS, Lin CL, Chung JD, Yang JC: **Postglacial population growth of *Cunninghamia konishii* (Cupressaceae) inferred from phylogeographical and mismatch analysis of chloroplast DNA variation.** *Mol Ecol* 2003, **12**:2689–2695.

Lu SY, Hung KH, Liu SL, Cheng YP, Wu WL, Chiang TY: **Genetic variation and population differentiation of *Michelia formosana* (Magnoliaceae) based on cpDNA variation and RAPD fingerprints: relevance to post-Pleistocene recolonization.** *J Plant Res* 2002, **115**:203–216.

Shih FL, Hwang SY, Cheng YP, Lee PF, Lin TP: **Uniform genetic diversity, low differentiation, and neutral evolution characterize contemporary refuge population of Taiwan fir (*Abies kawakami*, Pinaceae).** *Am J Bot* 2007, **94**:194–202.

Wu SH, Hwang CY, Lin TP, Chung JD, Cheng YP, Hwang SY: **Contrasting of phylogeographical pattern of two closely related species, *Machilus thubergii* and *Machilus kusanoi* (Lauraceae), in Taiwan.** *J Biogeogr* 2006, **33**:936–947.

Wu MJ, Huang SF, Huang TC, Li PF, Lin TP: **Evolution of the *Euphrasia transmorrisonensis* complex (Orobanchaceae) in alpine areas of Taiwan.** *J Biogeogr* 2005, **32**:1921–2929.
