# Supplementary material for: Multilocus Analyses Reveal Postglacial Demographic Shrinkage of Juniperus morrisonicola (Cupressaceae), a Dominant Alpine Species in Taiwan
Source: PLoS One. 2016 Aug 25;11(8):e0161713. doi: 10.1371/journal.pone.0161713 (PMC4999204; doi:10.1371/journal.pone.0161713)
Supplement: S4 Table — (PDF) [file pone.0161713.s010.pdf]

S4 Table Prior distributions and summary statistics used in DIYABC analyses.

(A) Sequence data

Parameters	Prior distribution	Constraints
N1	uniform [10–50000]	
N2	uniform [10–50000]	
Na	uniform [10–10000000]	Na > N1
N2a	uniform [10–50000]	N2a < N2
t1	uniform [10–50000]	t1 > t2
t2	uniform [10–50000]	t2 > t3
t3	uniform [10–50000]	t3 > t4
t4	uniform [10–50000]	

Summary Statistics
One sample
Number of haplotypes
Number of segregating sites
Mean of pairwise difference
Tajima's D
Private segregating sites
Two samples
Number of haplotypes
Numer of segregating sites

(B) Microsatellites

Parameters	Prior distribution	Constraints
N1	uniform [10–20000]	
N2	uniform [10–20000]	
Na	uniform [10–10000000]	Na > N1
N2a	uniform [10–20000]	N2a < N2
t1	uniform [10–50000]	t1 > t2
t2	uniform [10–50000]	t2 > t3
t3	uniform [10–50000]	t3 > t4
t4	uniform [10–50000]	

Summary Statistics
One sample
Mean number of alleles
Mean genic diversity
Mean size variance
Mean Garza-Williamson's M
Two samples
Mean number of alleles
Mean genic diversity
Mean size variance
