# Supplementary material for: Multilocus Analyses Reveal Postglacial Demographic Shrinkage of Juniperus morrisonicola (Cupressaceae), a Dominant Alpine Species in Taiwan
Source: PLoS One. 2016 Aug 25;11(8):e0161713. doi: 10.1371/journal.pone.0161713 (PMC4999204; doi:10.1371/journal.pone.0161713)
Supplement: S5 Table — (PDF) [file pone.0161713.s011.pdf]

(A) trnS-trnG

Haplotypes	DB	SS	HH	HK	KS	NH	SC	TK	YS	JF	JC
TS_1	9	7	7	8	5	8	9	1	6		
TS_2				1	6		6	4	2		
TS_3							1	1			
TS_4								1	1		
TS_5											1
TS_6										2	

(B) trnT-trnL

Haplotypes	DB	SS	HH	HK	KS	NH	SC	TK	YS	JF	JC
TT_1	11	6	8	8	11	11	9	7	7	1	
TT_2		1	1								
TT_3											1
TT_4										1	

(C) coxI

Haplotypes	DB	SS	HH	HK	KS	NH	SC	TK	YS	JF	JC
Co1_1	10	5	10	9	9	12	7	6	6	1	1
Co1_2	1	2					1	1			
Co1_3										1	

(D) coxIII

Haplotypes	DB	SS	HH	HK	KS	NH	SC	TK	YS	JF	JC
Co3_1	9	7	9	8	10	13	17	7	9	1	
Co3_2	1			1							
Co3_3											1
Co3_4										1	

(E) Chs

Haplotypes	DB	SS	HH	HK	KS	NH	SC	TK	YS	JF	JC
C_01	22	13	20	20	24	29	40	19	19	1	1
C_02					1				1		
C_03	3					1			1		
C_04	2										
C_05	1										
C_06	1										
C_07	1			1			1				
C_08	2										
C_09	1		1			1					
C_10		1		1							
C_11		3									
C_12		1									
C_13		2									
C_14		1						1			
C_15			1		1						
C_16			2								
C_17			2								
C_18			1								
C_19				4	2		2				
C_20				2							
C_21				2							
C_22					1			1			
C_23					1						
C_24					1						
C_25						1					
C_26						2			1		
C_27						1					
C_28							2				
C_29							2				
C_30							2				
C_31							1				
C_32							1				
C_33									2		
C_34											1
C_35										1	
C_36										1	

(F) Maldehy

Haplotypes	DB	SS	HH	HK	KS	NH	SC	TK	YS	JF	JC
Ma_01	1										
Ma_02	1										
Ma_03	13	1	3	2	1	1	9	12	2		1
Ma_04	7	5	13	13	8	15	18	4	11	1	1
Ma_05	2		3	3			1				1
Ma_06	2								1		
Ma_07	1					3					
Ma_08	1										
Ma_09	2				1	1	1				
Ma_10		1	1				1				
Ma_11		1				1					
Ma_12		1					3				
Ma_13			1								
Ma_14			1								
Ma_15			1					1			
Ma_16			1								
Ma_17			1								
Ma_18				1							
Ma_19				1							
Ma_20				1							
Ma_21				1							
Ma_22				2			2		2		
Ma_23					1						
Ma_24						1					
Ma_25						1					
Ma_26							2				
Ma_27							2				
Ma_28							1				
Ma_29							1				
Ma_30							1				
Ma_31							1				
Ma_32							2				
Ma_33							3				
Ma_34								1			
Ma_35								1			
Ma_36								1			
Ma_37									2		
Ma_38									1		
Ma_39									1		
Ma_40										2	
Ma_41					1						

(G) Myb

Haplotypes	DB	SS	HH	HK	KS	NH	SC	TK	YS	JF	JC
My_01	2	18									2
My_02	6	2	7	7	6	10	9	8	7		
My_03	3				2						
My_04	2			3		2					
My_05	15		9	10	10	19	15	4	5		
My_06	1					4					
My_07	3				1						
My_08	1		1	2	1			1			
My_09		1									
My_10			3								
My_11			2		1		1				
My_12			2								
My_13			2				3				
My_14			1								
My_15			1								
My_16			1								
My_17			1								
My_18				2							
My_19				1							
My_20				2							
My_21					2						
My_22					2				1		
My_23					5						
My_24					1						
My_25						2					
My_26						1		1			
My_27						1					
My_28							2				
My_29							2				
My_30							2				
My_31							2				
My_32							1				
My_33							1				
My_34							1				
My_35							1				
My_36							2				
My_37							1				
My_38							2				
My_39							1				
My_40								2			
My_41								2			
My_42								1			
My_43								2			
My_44									3		

My_45									1	
My_46									1	
My_47										2
My_48										1
My_49										1
My_50	2									
My_51	2									
My_52				1						
My_53							1			
My_54		2								

(H) Needly

Haplotypes	DB	SS	HH	HK	KS	NH	SC	TK	YS	JF	JC
N_01	2										
N_02	1										
N_03	6	2				1	1	3			
N_04	9	3	8	8	16	9	7	3	9		
N_05	1										
N_06	5		3	6	2	2	7	1	2		
N_07	1			2		2			1		
N_08	1		1						1		
N_09	2		1			4		3			
N_10	1		2	7	8	5	3	8	5		
N_11	3										
N_12	1		1			2					
N_13		13	5	3			4	1			3
N_14			2								
N_15			2								
N_16			1								
N_17			1								
N_18			1				1				
N_19			1								
N_20			1								
N_21				1			1				
N_22					1						
N_23						2					
N_24		1				1					
N_25						1					
N_26						3					
N_27						1					
N_28							2				
N_29							2				
N_30							1				
N_31							2				
N_32							1				
N_33							2				
N_34							1				
N_35							1				
N_36									2		
N_37									1		
N_38										5	
N_39										1	
N_40				2							
N_41				1							
N_42					1						
N_43						1					
N_44						1					

N_45	1	
N_46		1

(I) Pgi

Haplotypes	DB	SS	HH	HK	KS	NH	SC	TK	YS	JF	JC
P_01	3	2	8	2	3	4	10	2	3		
P_02	5	1	1	3	6	8	4	4	8		1
P_03	14	7	7	8	11	11	5	1	5		
P_04	1		1	2			1		1		
P_05	6	5	5	12	3	5	10	2	4		1
P_06	1										
P_07	1					1					
P_08	1			1							
P_09	1										
P_10							1				
P_11		1									
P_12		2									
P_13			3		1	4		2	2		
P_14			1								
P_15				1							
P_16				1			3	4			
P_17				1		2	6				
P_18					1						
P_19					1		5	3			
P_20					1						
P_21					1						
P_22					1						
P_23					1			1			
P_24						2					
P_25						1		1			
P_26						1					
P_27							3				
P_28							1				
P_29		2									
P_30									1		
P_31									1		
P_32										2	
P_33						1					
P_34						1					
P_35						1					
P_36							2				
P_37							1				
P_38							1				
P_39							1				
P_40									2		
